# IoT-Driven Robust Bearing Fault Diagnosis for Induction Motors Under Operating-Condition Shift

**DOI:** 10.3390/s26123829

**Published:** 2026-06-16

**Authors:** Şükrü Mustafa Kaya, Alireza Esmaeili Jobani

**Affiliations:** 1 Department of Computer Technologies, Blockchain Application Research Centre, Istanbul Aydin University, Istanbul 34295, Türkiye; 2 Department of Computer Engineering, Istanbul Aydin University, Istanbul 34295, Türkiye; alirezajobani@stu.aydin.edu.tr

**Keywords:** Industrial Internet of Things (IIoT), induction motor, bearing fault diagnosis, condition monitoring, operating-condition shift, vibration–current fusion

## Abstract

**Highlights:**

1. A controlled comparative evaluation is conducted for induction motor bearing diagnosis under operating-condition shift.
2. Raw temporal 1D-CNN, STFT-based 2D-CNN, and vibration–current fusion strategies are compared under the same preprocessing and validation protocol.
3. Measurement-wise and operating-condition holdout results are compared to show the effect of validation protocol on reported diagnostic robustness.
4. Simple vibration–current early fusion provides the most favorable robustness–complexity trade-off under operating-condition shift, while stricter bearing-code-disjoint testing reveals the remaining difficulty of unseen bearing-identity generalization.
5. The selected model remains compact with 73,891 parameters; embedded-hardware validation is identified as future work.

**Abstract:**

Reliable bearing fault diagnosis in induction motors is essential for predictive maintenance and Industrial Internet of Things (IIoT) applications. However, diagnostic models that perform well under random or measurement-wise data splits may fail when deployed under unseen operating conditions. This study presents a robustness-oriented comparative evaluation of induction motor bearing fault diagnosis models using vibration and phase-current signals from a controlled medium subset of the Paderborn bearing dataset. Raw temporal 1D-CNN models, STFT-based 2D-CNN representations, and vibration–current fusion strategies were evaluated under measurement-wise and operating-condition holdout protocols. Under measurement-wise validation, the 1D-CNN Early Fusion model achieved a Macro-F1 score of 0.9251. Under the stricter operating-condition holdout setting, the same model achieved the highest robustness among the evaluated CNN models. Multi-seed validation confirmed its stability, with a mean Macro-F1 of 0.8626, a worst-case Macro-F1 of 0.7159, and a robustness score of 0.7850. The selected model remained lightweight, requiring 73,891 trainable parameters and an estimated model size of 0.282 MB. Additional revision experiments were conducted to address bearing-identity sharing and classical baseline comparisons. In the bearing-code-disjoint validation test, both raw temporal models showed reduced performance, and early fusion did not significantly outperform vibration-only learning. The 1D-CNN Vibration model achieved a mean Macro-F1 of 0.5616, while the 1D-CNN Early Fusion model achieved 0.5485; the paired Wilcoxon test was not significant (*p* = 0.2016). Classical baselines using handcrafted time-domain, frequency-domain, envelope-inspired, and spectral-kurtosis features were also evaluated. The strongest classical baseline, vibration-feature XGBoost, achieved a mean Macro-F1 of 0.8582 under condition-holdout validation. Overall, the findings show that lightweight vibration–current early fusion provides a favorable robustness–complexity trade-off under operating-condition shift. However, the bearing-code-disjoint results indicate that complete generalization to unseen bearing identities remains challenging. Therefore, the deployment claims are limited to computational feasibility indicators, and further validation on embedded hardware, additional datasets, and stricter cross-domain protocols is required.

## 1. Introduction

Rolling-element bearings are among the most critical components in induction motors and electrical drive systems. Bearing degradation may lead to increased vibration, abnormal current signatures, reduced efficiency, secondary mechanical damage, and unexpected shutdowns. Therefore, reliable bearing fault diagnosis is an essential part of condition monitoring and predictive maintenance in electrical machines. In recent years, deep learning has become a dominant approach in this field because it can learn fault-related representations directly from measured signals and reduce the dependence on handcrafted feature extraction. Several review studies have confirmed the rapid growth of deep learning-based bearing fault diagnosis, including CNN-based diagnosis, transfer learning, vibration-based diagnosis, and fault detection in electrical machines [1,2,3,4,5].

Despite this progress, developing diagnostic models that remain reliable under variable operating conditions is still challenging. Many deep learning models report high classification accuracy when training and testing samples are randomly divided or when similar operating conditions appear in both training and testing sets. However, such evaluation protocols may overestimate real diagnostic performance because they do not fully represent deployment scenarios where the motor may operate under unseen speed, load, or radial force conditions. This issue is particularly important for induction motor monitoring, where the signal distribution can change significantly across operating regimes. Therefore, diagnostic models should be assessed not only by average accuracy but also by their ability to generalize across unseen operating conditions.

CNN-based approaches have shown strong performance in bearing fault diagnosis. Zhang et al. proposed a deep CNN model for bearing fault diagnosis under noisy environments and different working loads, showing that convolutional models can learn robust discriminative patterns from fault signals [6]. Eren et al. developed a compact adaptive 1D-CNN classifier for intelligent bearing fault diagnosis and demonstrated the suitability of one-dimensional temporal convolution for raw signal classification [7]. Lightweight and frequency-domain 1D-CNN models have also been proposed to improve robustness while reducing computational cost [8]. These studies show that 1D-CNN models are effective for rotating machinery diagnosis, especially when diagnostic information is preserved in the temporal or frequency structure of the input signal.

In addition to raw one-dimensional learning, time–frequency and image-based representations have been widely investigated. Vibration signals can be transformed into two-dimensional images using vibration imaging, wavelet packet transform, time–frequency maps, scalograms, or enhanced time–frequency representations. These representations allow CNNs to learn spatial patterns from transformed signal images. Hoang and Kang used vibration images for rolling element bearing fault diagnosis [9], while Islam and Kim proposed a wavelet-packet-transform-based 2D representation with a deep CNN [10]. Other studies have developed enhanced time–frequency CNNs, scalogram-based CNNs, and Gramian time–frequency enhancement networks for bearing fault diagnosis [11,12,13]. Although these methods can achieve high performance, they also introduce additional preprocessing steps and may increase computational complexity. Moreover, their superiority over raw 1D temporal learning is not always guaranteed under strict unseen-condition evaluation.

The choice of signal modality is another important issue. Vibration signals are widely used because localized bearing defects often produce mechanical impulses that are directly reflected in vibration measurements. However, induction motors also provide electrical measurements such as stator or phase current, which may already be available in industrial drive systems. Current-based diagnosis is attractive because it can reduce the need for additional sensors in some applications. Previous research has shown that motor current signals can contain useful diagnostic information for bearing fault diagnosis [14]. Nevertheless, current-based fault signatures may be weaker or more indirect than vibration-based signatures because bearing faults are primarily mechanical phenomena.

To improve diagnostic reliability, multi-source and multimodal fault diagnosis methods have been proposed. Current-based diagnosis and information fusion provide a practical basis for combining mechanical and electrical measurements in induction motor bearing diagnosis, while current-based deep learning approaches have also highlighted the potential of stator-current monitoring for bearing fault diagnosis [15]. Existing fusion strategies can be broadly categorized into input-level early fusion, feature-level fusion, and adaptive fusion mechanisms. Early fusion is simple and computationally efficient because multiple signals are combined before feature extraction. Feature-level fusion uses separate branches for each modality and combines learned representations before classification. Adaptive or gated fusion can learn modality weights, but it also increases model complexity. A key question is whether the additional complexity of feature-level or gated fusion improves cross-condition generalization, or whether a simpler Early Fusion model can provide a better robustness–complexity trade-off.

To address the above gaps, this study provides a controlled comparative analysis of induction motor bearing fault diagnosis models under operating-condition shift using vibration and phase-current signals from a medium subset of the Paderborn bearing dataset. The study does not claim novelty in the condition-holdout validation protocol itself or in the basic CNN and fusion architectures used. Instead, its contribution lies in evaluating raw temporal learning, STFT-based time–frequency learning, and vibration–current fusion strategies under the same preprocessing, validation, robustness, and complexity-analysis settings. This design allows the study to clarify which signal representation and fusion strategy offers the most reliable robustness–complexity trade-off under operating-condition shift.

The comparative evaluation is designed to address three practical limitations in existing bearing fault diagnosis studies. First, many studies report high diagnostic performance under random or measurement-wise data splits, which may overestimate real deployment performance because similar operating conditions can appear in both training and test sets. Second, although vibration-based diagnosis has been widely investigated, the standalone and auxiliary value of phase-current information remains less clear under strict unseen-condition testing. Third, peak accuracy alone does not capture worst-case performance, cross-condition variability, random-seed stability, model size, or inference efficiency.

The main contributions of this study are summarized as follows. First, a controlled medium subset of the Paderborn bearing dataset is constructed using 22 bearing codes, four operating conditions, and three diagnostic classes, including healthy, artificial damage, and real damage. Second, vibration-only, current-only, raw early-fusion, STFT-based 2D-CNN, dual-branch feature fusion, and gated fusion models are evaluated under the same preprocessing and validation protocols. Third, operating-condition holdout validation is used as a stricter evaluation setting than measurement-wise splitting, where each operating condition is excluded from training and used as an unseen test condition.

Fourth, robustness is evaluated using mean Macro-F1, standard deviation of Macro-F1, worst-case Macro-F1, and a robustness score. Fifth, multi-seed validation is performed for the two strongest raw temporal models to assess stability across random initializations.

Sixth, an additional bearing-code-disjoint validation experiment is conducted to examine whether the operating-condition holdout results are affected by bearing-identity sharing between training and test sets. In this stricter test, complete bearing codes are excluded from training and validation and used only for testing.

Seventh, classical machine-learning baselines are added using handcrafted time-domain, frequency-domain, envelope-inspired, and spectral-kurtosis features. Random Forest, Extra Trees, Linear SVM, and XGBoost classifiers are evaluated under both measurement-wise and operating-condition holdout protocols to provide a non-deep-learning comparison.

Finally, model complexity and inference time are analyzed to identify a practical robustness–complexity trade-off, while embedded-device deployment is left for future validation. The results show that the 1D-CNN Early Fusion model provides the most favorable robustness–complexity balance among the evaluated CNN models under operating-condition shift. However, the additional bearing-code-disjoint experiment shows that this conclusion should not be interpreted as complete generalization to unseen bearing identities.

## 2. Related Work

### 2.1. Current-Based, Multi-Source, and Multimodal Diagnosis

Current-based bearing fault diagnosis has received increasing attention in induction motor monitoring because electrical measurements are often available in industrial drive systems without requiring additional mechanical sensors. In this context, Guan et al. proposed a current-signal-based bearing fault diagnosis method using time shifting and CausalConvNets, showing that motor current signals can be enhanced to improve diagnostic representation [16]. This supports the view that current signals may contain useful fault-related information, although such information is usually more indirect than vibration-based fault signatures.

Beyond single-source current analysis, multi-source learning has been investigated to improve diagnostic reliability. Dong et al. proposed a motor bearing fault diagnosis method based on multi-source data and a lightweight one-dimensional CNN, demonstrating that compact deep models can benefit from complementary measurement sources [17]. Xue et al. developed a framework based on multi-transformation-domain and multi-source data for motor bearing fault diagnosis, indicating that different signal domains and sources can provide complementary diagnostic information [18].

Multimodal learning has also been explored under variable operating conditions. Alam et al. investigated multimodal bearing fault classification using vibration and motor phase-current signals with a 1D-CNN and transfer learning, emphasizing the importance of adapting diagnostic models to changing operating regimes [19]. Ayankoso et al. compared the performance of vibration and current signals in induction motor fault diagnosis using deep learning and machine-learning methods, showing that the diagnostic contribution of each modality can vary depending on the model and signal condition [20]. In addition, Jiménez-Guarneros et al. studied combined mechanical and electrical fault diagnosis in ASD-powered induction motors using MODWT and a lightweight 1D-CNN model [21].

These studies show that electrical measurements and multi-source learning can support bearing fault diagnosis in induction motors. However, the most effective way to combine vibration and phase-current signals under strict unseen operating-condition testing remains insufficiently clarified. In particular, it is still necessary to determine whether simple input-level fusion can provide better robustness–complexity balance than more complex feature-level or adaptive fusion strategies.

### 2.2. Benchmark Datasets and Operating-Condition Shift

Benchmark datasets play an important role in the development and comparison of data-driven bearing fault diagnosis methods. Among them, the Paderborn bearing dataset is particularly relevant for induction motor bearing diagnosis because it includes healthy bearings, artificially damaged bearings, and bearings with real damage under multiple operating conditions. In addition, the dataset provides both mechanical and electrical measurements, including vibration and motor current signals, which makes it suitable for evaluating single-modality and multimodal diagnostic models [22].

Operating-condition shift is one of the main challenges in practical bearing fault diagnosis. A model trained under one speed, load, or radial force condition may not perform reliably when tested under a different operating condition. This limitation is especially important for induction motor monitoring because real industrial systems rarely operate under fixed and fully controlled conditions. Therefore, diagnostic models should be evaluated not only under conventional random or measurement-wise splits, but also under stricter protocols that test their ability to generalize to unseen operating regimes.

Transfer learning has been widely investigated as one way to address domain shift in bearing fault diagnosis. Chen et al. reviewed deep transfer learning approaches for bearing fault diagnosis and emphasized that distribution differences between source and target domains remain a major challenge in practical diagnostic applications [23]. However, transfer learning often requires access to target-domain data or additional adaptation steps, which may not always be available in real deployment scenarios.

Recent domain-adaptation studies have also investigated explainable bearing fault diagnosis under varying operating conditions. Rezazadeh et al. proposed a prototype-attention domain adaptation framework that aligns class centroids and dynamically weights target samples based on similarity and confidence [24]. Their study was evaluated on benchmark bearing datasets, including Paderborn and CWRU, and highlights the relevance of domain-adaptation strategies for operating-condition variation. However, such methods typically introduce additional adaptation mechanisms, whereas the present study focuses on a controlled comparison of lightweight raw temporal, time–frequency, fusion-based, and classical baseline models without target-domain adaptation.

Many studies have also relied on widely used benchmark datasets such as the Case Western Reserve University dataset. Neupane and Seok reviewed deep learning-based bearing fault diagnosis studies using this dataset and pointed out that benchmark-based evaluations have contributed significantly to model development, but they may also encourage validation settings that do not fully represent real deployment conditions [25]. This highlights the need for evaluation protocols that go beyond random data splitting and directly test robustness under unseen operating conditions.

In this context, condition-holdout validation provides a stricter and more deployment-oriented evaluation strategy. Instead of randomly splitting samples, one complete operating condition is excluded from training and validation and used only for testing. This allows the diagnostic model to be assessed under an operating regime that was not observed during training. Therefore, the Paderborn dataset provides an appropriate experimental basis for the present study, where vibration-only, current-only, and vibration–current fusion models are compared under both measurement-wise and strict operating-condition holdout protocols.

### 2.3. Advanced Raw Temporal CNN Models

Raw temporal CNN-based models have been further developed through multi-scale feature extraction, recurrent extensions, optimized architectural design, and attention mechanisms. Huang et al. proposed an improved deep CNN with multi-scale information for bearing fault diagnosis, showing that extracting features at different temporal resolutions can improve the representation of fault-related signal patterns [26]. Chen et al. combined multi-scale CNN and LSTM modules to capture both local convolutional features and sequential dependencies in bearing signals [27]. These studies indicate that temporal deep learning models can be strengthened by considering both local signal structures and longer-range dependencies.

Other studies have focused on improving CNN architectures and model design for bearing fault classification. Magar et al. introduced FaultNet, a deep CNN architecture for bearing fault classification, demonstrating the effectiveness of deeper convolutional feature extraction for diagnostic tasks [28]. Ruan et al. investigated CNN parameter design based on fault signal analysis and emphasized that convolutional architecture should be aligned with the characteristics of bearing fault signals [29]. This highlights that model performance depends not only on network depth, but also on appropriate architectural choices related to the input signal.

Recent works have also explored hybrid and attention-enhanced CNN models under more challenging conditions. Song et al. proposed an optimized CNN-BiLSTM network for bearing fault diagnosis under multiple working conditions with limited training samples, showing the potential of combining convolutional and recurrent learning for condition-varying scenarios [30]. Huang et al. developed a multi-scale convolutional network with a channel attention mechanism for rolling bearing fault diagnosis, indicating that attention-based feature weighting can improve the extraction of discriminative fault information [31].

These studies demonstrate that raw temporal and CNN-based learning approaches remain highly effective for bearing fault diagnosis. However, many of them primarily emphasize classification accuracy or architectural improvement, while fewer studies jointly consider strict operating-condition holdout validation, worst-case robustness, random-seed stability, model size, and inference efficiency. Therefore, a deployment-oriented comparison is still needed to determine whether more complex temporal architectures are necessary, or whether a lightweight raw temporal fusion model can provide a more practical robustness–complexity trade-off.

### 2.4. Advanced Time–Frequency and Image-Based Diagnosis

Time–frequency and image-based representations have also been developed to improve the visibility of bearing fault patterns in transformed signal domains. Xu et al. proposed a hybrid deep learning model for rolling bearing fault diagnosis, combining deep feature extraction and classification mechanisms to improve diagnostic performance [32]. Ahmed and Nandi developed connected-components-based color image representations of vibration signals for two-stage roller bearing fault diagnosis using CNNs, showing that transformed visual representations can support fault discrimination [33].

Recent studies have further explored two-dimensional time–frequency representations and data augmentation strategies. Fu et al. proposed a rolling bearing fault diagnosis method based on 2D time–frequency images and data augmentation, demonstrating that transformed signal images can improve the diversity and discriminative capacity of training samples [34]. Kim and Kim investigated vibration spectrogram analysis using Grad-CAM-based feature selection and statistical analysis, highlighting the importance of interpretability in spectrogram-based bearing diagnosis [35].

Interpretable and noise-robust time–frequency learning has also attracted attention. Wang et al. proposed an interpretable convolutional neural network with multilayer wavelet representation for noise-robust machinery fault diagnosis, showing that wavelet-based feature representations can improve robustness under noisy conditions [36]. These studies indicate that time–frequency and image-based approaches can provide useful diagnostic representations, especially when fault-related spectral patterns are clearly expressed in the transformed domain.

However, time–frequency methods usually require additional preprocessing and may increase computational cost compared with raw temporal learning. Moreover, their superiority over raw 1D-CNN models is not always guaranteed when the test operating condition is completely unseen during training. Therefore, the present study directly compares raw temporal 1D-CNN models and STFT-based 2D-CNN models under the same condition-holdout validation protocol to determine which representation provides a more reliable robustness–complexity trade-off.

### 2.5. Sensor Fusion and Positioning of the Present Work

Sensor fusion has also been investigated in bearing fault diagnosis beyond the direct combination of vibration and motor current signals. Wang et al. proposed a bearing fault diagnosis approach based on vibro-acoustic data fusion and a 1D-CNN network, showing that complementary sensing information can improve diagnostic representation when different physical signal sources are jointly considered [37]. This supports the broader view that multi-sensor learning can be useful for machinery fault diagnosis, especially when each signal source captures different aspects of the fault mechanism.

However, the effectiveness of sensor fusion depends strongly on the type of signals, the fusion strategy, and the validation protocol. In practical motor monitoring, vibration signals directly reflect mechanical bearing defects, while phase-current signals may contain more indirect fault-related information through electromechanical coupling. Therefore, the key issue is not only whether multiple signals can be combined, but also how they should be combined under realistic operating-condition shifts.

Existing fusion approaches can generally be grouped into input-level early fusion, feature-level fusion, and adaptive or gated fusion. Early fusion combines raw signal channels before feature extraction and is computationally simple. Feature-level fusion uses separate branches to extract modality-specific representations before combining them. Adaptive or gated fusion introduces learnable weighting mechanisms to control the contribution of each modality. Although more advanced fusion methods may appear more flexible, they can also increase model complexity and may become more sensitive to condition-specific patterns in the training data.

The present study is positioned within this gap. Rather than proposing a deeper or more complex neural architecture, it systematically compares vibration-only, current-only, raw early-fusion, STFT-based 2D-CNN, dual-branch feature fusion, and gated fusion models under the same preprocessing, segmentation, and validation protocols. By evaluating these models under both measurement-wise and strict operating-condition holdout settings, the study aims to identify which representation and fusion strategy provides the most reliable robustness–complexity trade-off for induction motor bearing fault diagnosis.

As shown in Table 1, previous studies have made important contributions to vibration-based diagnosis, current-based diagnosis, time–frequency analysis, transfer learning, multi-source diagnosis, and robust fault classification. Nevertheless, many studies emphasize either a single signal modality, a specific representation, or learning transfer under domain shift. In contrast, the present study provides a deployment-oriented comparison of raw temporal learning, STFT-based learning, vibration–current fusion strategies, and classical feature-based baselines under measurement-wise, operating-condition holdout, and bearing-code-disjoint validation settings. The main objective is not to introduce a deeper architecture, but to determine which signal representation and fusion strategy provides the most reliable robustness–complexity trade-off under operating-condition shift, while also clarifying the remaining challenge of unseen bearing-identity generalization.

### 2.6. Research Gap

The reviewed literature indicates five main gaps. First, many studies still emphasize high accuracy under random, measurement-wise, or benchmark-specific settings, while fewer works evaluate strict operating-condition holdout generalization. Second, vibration-only, current-only, raw 1D-CNN, time–frequency 2D-CNN, and multimodal fusion strategies are often studied separately, making it difficult to determine which approach provides the best robustness–complexity trade-off. Third, current-based diagnosis is promising for induction motor monitoring, but its standalone and auxiliary value relative to vibration signals needs further systematic evaluation. Fourth, computational cost, inference time, model size, worst-case performance, and robustness variability are not always reported together with accuracy. Fifth, many studies do not jointly compare deep learning models with strong classical feature-engineering baselines under the same validation protocols.

This study addresses these gaps by comparing raw 1D-CNN models, STFT-based 2D-CNN models, several vibration–current fusion strategies, and classical feature-based machine-learning baselines under measurement-wise and operating-condition holdout protocols. In addition, an extra bearing-code-disjoint validation experiment is included to examine unseen bearing-identity generalization. Unlike studies that focus only on peak classification performance, this work evaluates mean Macro-F1, worst-case Macro-F1, robustness score, parameter count, model size, inference time, multi-seed stability, and paired statistical testing. This allows the diagnostic models to be assessed from both classification and practical induction motor condition-monitoring perspectives.

## 3. Materials and Methods

### 3.1. Overall Research Framework

**Figure 1:**
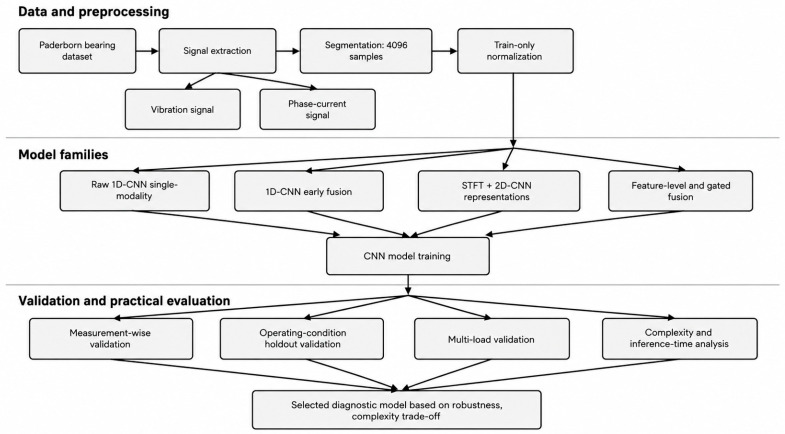
Overall workflow of the robustness-oriented comparative evaluation pipeline for induction motor bearing fault diagnosis. The framework compares raw temporal learning, STFT-based time–frequency learning, vibration–current fusion strategies, and classical feature-based baselines under measurement-wise, operating-condition holdout, and additional bearing-code-disjoint validation protocols.

This study develops a robustness-oriented comparative evaluation pipeline for induction motor bearing fault diagnosis under operating-condition shift. The evaluation pipeline compares raw temporal learning, time–frequency representation learning, and vibration–current fusion strategies using vibration and phase-current signals. Unlike conventional evaluation pipelines that mainly report classification accuracy under a single data split, the proposed framework evaluates diagnostic performance from three complementary perspectives: measurement-wise performance, cross-condition robustness, and computational practicality. The overall workflow of the comparative evaluation pipeline is shown in Figure 1. The pipeline includes signal extraction, segmentation, train-only normalization, model training, measurement-wise evaluation, condition-holdout evaluation, multi-seed validation, bearing-code-disjoint validation, classical baseline evaluation, and complexity analysis.

This framework was designed to evaluate not only classification performance, but also generalization under unseen operating conditions, cross-seed stability, model complexity, and inference efficiency.

The process starts with the extraction of vibration and phase-current signals from the Paderborn bearing dataset. The signals are segmented into fixed-length windows and normalized using only the training subset of each validation split. The resulting segments are then used to train and evaluate raw 1D-CNN models, STFT-based 2D-CNN models, and several vibration–current fusion variants. Finally, the models are compared using classification metrics, condition-holdout robustness metrics, multi-seed validation, and inference-time analysis.

The main purpose of this framework is to determine not only which model achieves the highest average classification performance, but also which model maintains stable behavior when the test operating condition is not observed during training. This is essential for practical induction motor condition monitoring because deployment conditions may differ from the operating regimes available in the training data.

### 3.2. Dataset Description

The experiments were conducted using the Paderborn bearing dataset, which was widely used in recent induction motor and motor-bearing fault diagnosis studies because it provides bearing health states under multiple operating conditions together with mechanical and electrical measurements [38,39]. The dataset is suitable for the present study because it includes healthy bearings, artificially damaged bearings, and bearings with real damage under multiple operating conditions. It also provides both mechanical and electrical measurements, including vibration and motor current signals.

In this work, a medium subset of the dataset was constructed to provide sufficient class and condition diversity while maintaining manageable computational cost. The subset contained 22 bearing codes, including 6 healthy bearing states, 8 artificially damaged bearing states, and 8 real-damage bearing states. Four operating conditions were used: N09_M07_F10, N15_M01_F10, N15_M07_F04, and N15_M07_F10. Each measurement file was represented by 10 non-overlapping signal segments, resulting in 17,590 total segments. Table 2 summarizes the medium Paderborn subset used in this study.

The medium subset was selected to preserve all three diagnostic classes and all four operating conditions while keeping the computational cost feasible for repeated condition-holdout and multi-seed experiments. The selected subset included six healthy bearing states, eight artificially damaged bearing states, and eight real-damage bearing states. Each valid measurement file was divided into 10 non-overlapping segments. One artificial-damage measurement file could not be parsed successfully and was therefore excluded during preprocessing, resulting in 17,590 valid segments in total.

The selected bearing codes, diagnostic classes, operating conditions, number of measurement files, and segment counts used in the medium subset are provided in Supplementary Table S2 to support reproducibility.

To further improve reproducibility, the revised Supplementary Material also provides the file-level manifest, split assignments, and excluded-file information. The selected bearing codes were K001–K006 for the healthy class; KA01, KA03, KA04, KA05, KA06, KA07, KA08, and KA09 for the artificial-damage class; and KI01, KI03, KI04, KI05, KI07, KI08, KI14, and KI16 for the real-damage class. One artificial-damage file, N15_M01_F10_KA08_2.mat, was excluded because it could not be parsed consistently during preprocessing. The final subset contained 1759 valid measurement files and 17,590 non-overlapping signal segments. The selected bearing-code composition and per-condition segment counts are reported in Supplementary Table S2a,b. To support reproducibility without overloading the Supplementary Document with very large tables, the complete file-level manifest, segment-level metadata, measurement-wise split assignments, and excluded-file information are provided as machine-readable Supplementary CSV Files.

This subset design allowed the models to be evaluated under both measurement-wise and operating-condition holdout protocols while maintaining comparable sample availability across the four operating conditions.

**Figure 2:**
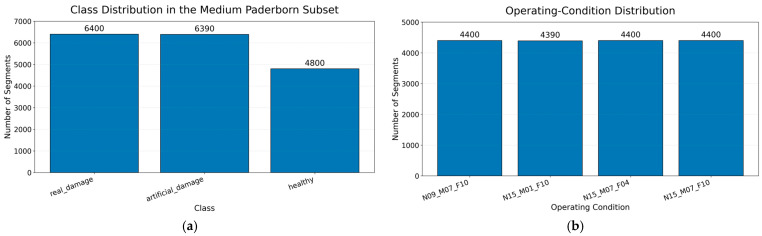
Distribution of the medium Paderborn subset: (**a**) class distribution; (**b**) operating-condition distribution.

The class distribution and operating-condition distribution of the medium Paderborn subset are shown in Figure 2. Figure 2a shows the class distribution of the medium Paderborn subset, while Figure 2b shows the number of segments available for each operating condition.

As shown in Figure 2, the selected subset is reasonably balanced across operating conditions, with approximately 4400 segments per condition. The class distribution includes 4800 healthy segments, 6390 artificial-damage segments, and 6400 real-damage segments. Although the healthy class contains fewer segments than the two faulty classes, the dataset still provides sufficient samples for evaluating healthy, artificial-damage, and real-damage bearing states.

The approximately balanced distribution across operating conditions makes the subset suitable for condition-holdout evaluation. Since each condition has a comparable number of segments, each operating condition can be excluded from training and used as an unseen test condition without creating a severely imbalanced test set. This structure allows the evaluation protocol to focus on operating-condition generalization rather than being dominated by differences in test-set size.

### 3.3. Signal Extraction, Segmentation, and Normalization

The phase-current signal was included as an auxiliary electrical modality because recent induction motor fault diagnosis and multi-source bearing diagnosis studies indicate that electrical measurements can provide complementary diagnostic information when combined with vibration-based sensing [40].

Each signal was divided into fixed-length segments of 4096 samples. Non-overlapping segmentation was used to reduce redundancy between neighboring samples and to keep the computational cost manageable. From each valid measurement file, 10 non-overlapping segments were extracted. The segment start and end indices for all extracted windows are provided in the machine-readable segment-level metadata file described in Supplementary Table S2c.

This strategy allowed the dataset to preserve representative information from each measurement file while avoiding excessive repetition of highly similar signal windows.

For each experimental split, normalization was performed using only the training subset. The mean and standard deviation were computed from the training data and then applied to the validation and test sets. This procedure was applied separately to vibration and current signals. Using training-only normalization is important because it prevents information from validation or test data from influencing the preprocessing stage, thereby reducing the risk of data leakage [41].

After segmentation and normalization, each vibration input segment was represented as a one-dimensional array of length 4096. The current signal was prepared using the same window length. For raw 1D-CNN early fusion, vibration and current segments were combined as two input channels. For single-modality models, only one signal channel was used. For STFT-based models, the same segmented signals were transformed into time–frequency representations before being used as inputs for the 2D-CNN models.

### 3.4. Experimental Validation Protocols

Two main validation protocols were used in this study: measurement-wise validation and operating-condition holdout validation. Following reviewer feedback, two additional evaluation components were included in the revised study: a bearing-code-disjoint validation experiment and a classical machine-learning baseline evaluation. The bearing-code-disjoint experiment was designed to test whether diagnostic performance remains stable when the bearing identities in the test set are completely unseen during training and validation. The classical baseline experiment was added to compare the CNN-based models with conventional handcrafted-feature-based diagnostic pipelines.

Measurement-wise validation was used to establish a baseline performance under a controlled split, while condition-holdout validation was used to evaluate model robustness under unseen operating conditions. In addition, multi-seed validation was conducted for the two strongest raw temporal models, namely the 1D-CNN Vibration and 1D-CNN Early Fusion models, to assess stability across random initializations and to verify whether the final model-selection decision remained consistent across different random seeds.

#### 3.4.1. Measurement-Wise Validation

Measurement-wise validation was used as the baseline evaluation protocol. In this protocol, measurement files were divided into training, validation, and test sets using group-based splitting at the measurement-file level. The split contained 1125 measurement files for training, 282 measurement files for validation, and 352 measurement files for testing, corresponding to 63.96%, 16.03%, and 20.01% of the dataset, respectively. Since each valid measurement file contributed 10 non-overlapping segments, this resulted in 11,250 training segments, 2820 validation segments, and 3520 test segments. All segments extracted from the same measurement file were kept in the same split. This prevented direct segment-level leakage between training, validation, and test sets. The exact measurement-wise split assignment for each segment and source measurement file is provided in the machine-readable segment-level metadata file described in Supplementary Table S2c.

This protocol evaluates whether the models can classify unseen measurement files when all operating conditions are represented across the data splits. Therefore, it is useful for establishing baseline diagnostic performance. However, since the same operating conditions are still present in training and testing, this protocol does not fully represent deployment scenarios where the model may encounter unseen operating regimes.

#### 3.4.2. Operating-Condition Holdout Validation

Operating-condition holdout validation was used to evaluate cross-condition generalization. In this protocol, one operating condition was completely excluded from training and validation and used only as the test condition. The remaining three operating conditions were used for model training and validation. This process was repeated four times so that each operating condition served once as the unseen test condition. Table 3 summarizes the operating-condition holdout validation protocol, including the holdout test condition and the corresponding training conditions for each evaluation setting.

This protocol is more challenging than measurement-wise validation because the test data are collected under an operating condition that is not observed during training. Therefore, it provides a stricter estimate of diagnostic robustness under operating-condition shift. This is important because recent domain generalization and unseen-condition fault diagnosis studies have shown that bearing diagnosis models can suffer performance degradation when load, speed, or working-condition distributions differ between training and testing domains [42,43,44]. This protocol evaluates generalization to unseen operating conditions rather than to unseen bearing identities; therefore, the same bearing code may appear under other operating conditions in the training set.

#### 3.4.3. Multi-Seed Validation

After the main condition-holdout experiments, multi-seed validation was applied to the two strongest raw temporal models, namely the 1D-CNN Vibration and 1D-CNN Early Fusion models. These two models were selected for additional stability analysis because they achieved the highest robustness scores among the evaluated lightweight raw temporal models in the condition-holdout experiments.

Each model was trained and evaluated using three random seeds: 42, 123, and 2024. For each random seed, both models were evaluated under all four operating-condition holdout settings. This procedure was used to determine whether the observed performance differences were stable across random initializations rather than being dependent on a single training run.

The final multi-seed results were reported using mean accuracy, standard deviation of accuracy, mean Macro-F1, standard deviation of Macro-F1, worst-case Macro-F1, weighted F1, and robustness score. This provided a more reliable estimate of model stability under unseen operating conditions and supported the final selection of the 1D-CNN Early Fusion model.

For each seed, both the model initialization and the group-based validation split within the non-holdout operating conditions were repeated. The holdout test condition itself remained unchanged, while the training and validation subsets were re-sampled from the remaining three operating conditions using the corresponding seed. Therefore, the multi-seed experiment evaluated sensitivity to both training stochasticity and validation-split variation, without allowing any samples from the holdout operating condition to enter training or validation.

It should be noted that this multi-seed analysis refers to the main operating-condition holdout comparison of the two strongest raw temporal CNN models. The additional bearing-code-disjoint validation described in Section 3.4.4 used five random seeds and was analyzed separately with paired statistical testing.

#### 3.4.4. Bearing-Code-Disjoint Validation Protocol 

To address the possibility that the operating-condition holdout protocol may still share bearing-specific signatures between training and test sets, an additional bearing-code-disjoint validation experiment was conducted. In this stricter setting, the test set contained bearing codes that were completely excluded from both training and validation. Therefore, unlike the operating-condition holdout protocol, this experiment evaluated generalization to unseen bearing identities.

Eight holdout scenarios were defined. In each scenario, one healthy bearing code, one artificial-damage bearing code, and one real-damage bearing code were removed from the training and validation data and used exclusively for testing. The remaining bearing codes were used for training and validation using a file-level train–validation split. Each scenario was repeated using five random seeds: 42, 123, 2024, 7, and 99. The two strongest raw temporal models, namely 1D-CNN Vibration and 1D-CNN Early Fusion, were evaluated in this experiment, resulting in 40 paired runs per model and 80 total model-training runs.

The same training-only normalization, model architecture, optimizer, early stopping strategy, and evaluation metrics used in the main experiments were applied. A paired Wilcoxon signed-rank test and a bootstrap confidence interval were used to compare the Macro-F1 differences between the 1D-CNN Early Fusion and 1D-CNN Vibration models across the 40 paired runs. This experiment was intended as a strict stress test of bearing-identity generalization and was not used to replace the operating-condition holdout evaluation.

### 3.5. CNN-Based Diagnostic Models

Several CNN-based diagnostic architectures were developed and compared to evaluate the effects of signal modality, input representation, and fusion strategy. The evaluated models included raw 1D-CNN models, STFT-based 2D-CNN models, dual-branch feature fusion, and gated vibration–current fusion. All models were trained for the same three-class diagnostic task, including healthy, artificial-damage, and real-damage classes.

The final raw 1D-CNN backbone consisted of three convolutional blocks. The first, second, and third Conv1D layers used 32, 64, and 128 filters, respectively, with kernel sizes of 9, 7, and 5. All convolutional layers used the same padding. Each convolutional layer was followed by batch normalization, ReLU activation, and max-pooling with a pool size of 2. The extracted feature maps were then passed through global average pooling, a dense layer with 128 units, dropout with a rate of 0.30, and a three-neuron softmax output layer.

For single-modality models, the input shape was 4096 × 1. For the raw 1D-CNN Early Fusion model, vibration and phase-current signals were stacked as two input channels, resulting in an input shape of 4096 × 2. The selected 1D-CNN Early Fusion model contained 73,891 trainable parameters, while the corresponding vibration-only model contained 73,603 trainable parameters.

For STFT-based models, each 4096-sample segment was first converted into a log-magnitude time–frequency representation. The resulting time–frequency maps were processed using a 2D-CNN architecture with three convolutional blocks. The first, second, and third Conv2D layers used 32, 64, and 128 filters, respectively, with 3 × 3 kernels, the same padding, and ReLU activation. Batch normalization was applied after each convolutional layer, and max-pooling with a 2 × 2 pool size was used after the first two convolutional blocks.

The Dual-Branch Feature Fusion model used two parallel raw 1D-CNN branches, one for vibration and one for phase current. Each branch extracted modality-specific temporal features before feature concatenation. The concatenated feature vector was then passed to the classification stage. This architecture was included to examine whether separate feature extraction for each modality improves cross-condition generalization compared with simple input-level fusion.

The gated fusion CNN also used separate vibration and phase-current branches, but introduced a learnable gating mechanism to adaptively weight modality-specific features before final classification. This model was evaluated to determine whether adaptive fusion improves robustness under unseen operating conditions.

Table 4 summarizes the evaluated diagnostic architectures, their input representations, fusion mechanisms, and computational characteristics.

**Figure 3:**
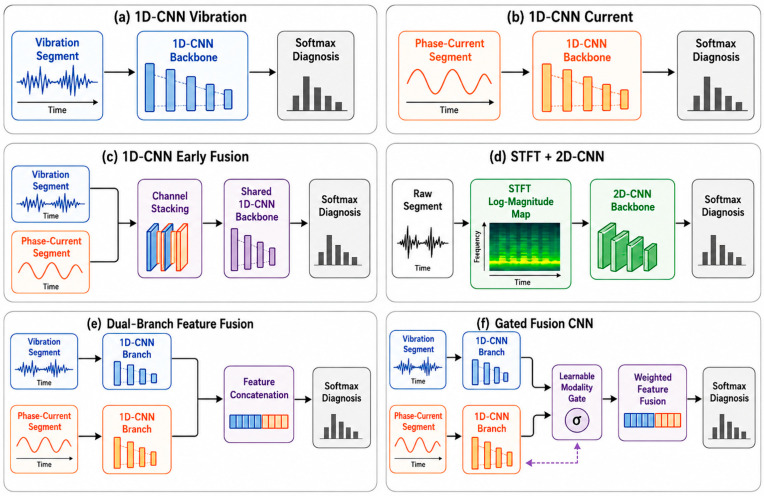
Schematic comparison of the evaluated CNN-based diagnostic architectures and fusion strategies.

To make the architectural differences between the evaluated single-modality, early-fusion, time–frequency, dual-branch, and gated-fusion models clearer, a schematic comparison of the model families is provided in Figure 3.

As illustrated in Figure 3, the evaluated models differ mainly in the input representation and the stage at which vibration and current information are combined. The purpose of comparing these architectures was not only to identify the highest-performing model, but also to determine whether additional architectural complexity is justified under strict operating-condition holdout validation. Therefore, all models were evaluated using the same dataset subset, segmentation strategy, normalization procedure, validation protocols, and performance metrics. This design enabled a fair comparison between raw temporal learning, time–frequency learning, simple early fusion, feature-level fusion, and adaptive gated fusion.

### 3.6. STFT Representation

For the STFT-based models, each 4096-sample segment was transformed into a time–frequency representation using the short-time Fourier transform. The STFT was computed with a frame length of 256 samples, a frame step of 128 samples, an FFT length of 256, and a Hann window. The magnitude spectrum was then converted into logarithmic scale as log(|STFT| + 10^−6^). This logarithmic magnitude representation was used to reduce the dominance of high-energy spectral components and to make weaker time–frequency structures more visible.

For the vibration-only STFT model, the vibration signal was transformed into a single-channel time–frequency map. For the vibration–current STFT early fusion model, the vibration and phase-current signals were transformed separately using the same STFT configuration, and the resulting log-magnitude maps were stacked as two input channels. This ensured that both modalities were represented under the same time–frequency resolution.

The STFT-based 2D-CNN architecture consisted of three convolutional blocks. The first, second, and third Conv2D layers used 32, 64, and 128 filters, respectively, with 3 × 3 kernels, the same padding, and ReLU activation. Batch normalization was applied after each convolutional layer, and max-pooling with a 2 × 2 pool size was used after the first two convolutional blocks. The extracted feature maps were then passed to the classification stage and mapped to the three diagnostic classes using a softmax output layer.

### 3.7. Training Configuration

All models were trained using mini-batch learning with a batch size of 128. The input pipeline was implemented using TensorFlow datasets with batching and automatic prefetching. For shuffled training datasets, the shuffle buffer size was set to the smaller value between the number of training samples and 5000. The Adam optimizer was used with an initial learning rate of 0.001.

For each experimental split, normalization parameters were computed only from the training subset. The mean and standard deviation of the training data were then applied to the corresponding validation and test sets. This procedure was used to prevent information leakage from validation or test samples into the preprocessing stage.

In the single-seed condition-holdout experiments, one operating condition was used exclusively as the test set, while the remaining three operating conditions were used for training and validation. The validation subset was obtained from the non-holdout conditions using group-based splitting with a validation ratio of 0.15 and a fixed random state of 42. The measurement file name was used as the grouping variable so that all segments extracted from the same measurement file remained in the same partition.

In the multi-seed experiments, the same group-based splitting procedure was repeated using the corresponding seed value. Therefore, both model initialization and the training–validation partition varied across seeds, while the holdout test condition remained unchanged and was never used during training or validation.

For the raw 1D-CNN models, the single-modality inputs had a shape of 4096 × 1, while the early-fusion input had a shape of 4096 × 2 by stacking vibration and phase-current signals as two channels. The selected 1D-CNN Early Fusion model contained 73,891 parameters.

For the raw 1D-CNN models, Dual-Branch Feature Fusion model, and gated fusion CNN, the maximum number of training epochs was set to 25. For the STFT-based 2D-CNN models, the maximum number of epochs was set to 15 to control the additional computational cost introduced by the time–frequency representation.

The model and training hyperparameters were fixed before final test-set evaluation and were not tuned using any holdout test condition. Architectural choices, including the number of convolutional blocks, filter sizes, kernel sizes, dropout rate, batch size, optimizer, learning rate, and STFT parameters, were selected based on preliminary validation behavior, consistency with lightweight CNN designs in the bearing-diagnosis literature, and computational feasibility. In all validation protocols, test data were used only once for final performance reporting and were never used for hyperparameter selection, early stopping, normalization, or model selection.

All experiments were conducted in Google Colab (Google LLC, Mountain View, CA, USA) using an NVIDIA Tesla T4 GPU with 15,360 MiB of memory (NVIDIA Corporation, Santa Clara, CA, USA). The software environment included Python 3.12.13 (Python Software Foundation, Wilmington, DE, USA), TensorFlow 2.20.0 with the Keras API (Google LLC, Mountain View, CA, USA), NumPy 2.0.2 (NumPy Developers), and XGBoost for the gradient-boosting baseline. The Random Forest, Extra Trees, and Linear SVM baselines were implemented using standard Python machine-learning libraries.

### 3.8. Evaluation Metrics

The diagnostic models were evaluated using accuracy, class-wise precision, class-wise recall, F1-score, Macro-F1, weighted F1, and confusion matrices. Since the diagnosis problem includes three classes, namely healthy, artificial damage, and real damage, Macro-F1 was selected as the primary performance metric. Macro-F1 gives equal importance to all classes and is therefore more informative than accuracy when class distributions are not perfectly balanced.

Let C denote the confusion matrix, where $C_{ij}$ represents the number of samples whose true class is i and predicted class is j. The overall classification accuracy was calculated as:

(1) $$Accuracy=\frac{\sum_{i=1}^{K}C_{ii}}{\sum_{i=1}^{K}\sum_{j=1}^{K}C_{ij}}$$

where K is the number of diagnostic classes. In this study, ***K*** = **3**.

For each class i, precision and recall were calculated using a one-vs-rest formulation:

(2) $$Precision_{i}=\frac{TP_{i}}{TP_{i}+FP_{i}}$$

(3) $$Recall_{i}=\frac{TP_{i}}{TP_{i}+FN_{i}}$$

where $TP_{i}$, $FP_{i}$, and $FN_{i}$ represent the true positives, false positives, and false negatives for class i, respectively. The F1-score for each class was calculated as the harmonic mean of precision and recall:

(4) $$F1_{i}=2\times \frac{Precision_{i}\times Recall_{i}}{Precision_{i}+Recall_{i}}$$

Macro-F1 was obtained by averaging the class-wise F1-scores:

(5) $$Macro\text{-}F1=\frac{1}{K}\sum_{i=1}^{K}F1_{i}$$

Weighted F1 was also reported to account for class support:

(6) $$Weighted\text{-}F1=\sum_{i=1}^{K}\frac{n_{i}}{N}F1_{i}$$

where $n_{i}$ is the number of samples in class i, and N is the total number of samples.

In addition to conventional classification metrics, robustness-oriented metrics were used to evaluate model stability across unseen operating conditions. For each model, the mean Macro-F1, standard deviation of Macro-F1, and worst-case Macro-F1 were calculated across the four condition-holdout tests. The robustness score was defined as:

(7) Robustness Score=Mean(Macro-F1)−Std(Macro-F1)

A higher robustness score indicates that a model achieves high average diagnostic performance while maintaining low variability across operating-condition shifts. This metric was used to support model selection under unseen-condition testing, together with worst-case Macro-F1, parameter count, model size, and inference time. The robustness score was used as a practical ranking criterion for comparing models under operating-condition shift, not as a replacement for the individual reporting of mean Macro-F1, standard deviation, and worst-case Macro-F1. Therefore, all three components were reported separately, together with the robustness score.

It should also be emphasized that the robustness score is a descriptive and heuristic stability indicator rather than a theoretically complete evaluation metric. It was introduced to summarize the trade-off between average Macro-F1 performance and variability across unseen operating conditions in a compact way. Therefore, it should not be interpreted as a standalone measure of diagnostic quality or as a substitute for Macro-F1, worst-case Macro-F1, class-wise F1, confidence intervals, or statistical testing. Model ranking in this study is therefore discussed using the robustness score together with mean performance, worst-case performance, class-wise behavior, model complexity, and the additional statistical analysis reported for the bearing-code-disjoint experiment.

### 3.9. Complexity and Inference-Time Analysis

In addition to diagnostic performance, computational complexity and inference efficiency were analyzed to assess the practical applicability of the evaluated diagnostic models. For each model, the number of trainable parameters, estimated model size, average inference time per segment, inference-time standard deviation, and throughput in segments per second were measured.

The number of trainable parameters was obtained directly from the corresponding Keras model. The estimated model size was calculated based on the trainable parameter count and the stored model representation. Inference time was measured using trained models and representative test segments under the same hardware and software environment. All measurements were performed in Google Colab using an NVIDIA Tesla T4 GPU with 15,360 MiB of memory. The software environment included Python 3.12.13, TensorFlow 2.20.0, and NumPy 2.0.2.

For inference-time measurement, the same input representation used during model evaluation was passed to each trained model. For raw 1D-CNN models, the input consisted of normalized time-domain signal segments. For STFT-based 2D-CNN models, the STFT transformation was implemented inside the model graph using TensorFlow operations. Therefore, the reported inference time for STFT-based models includes the time–frequency transformation and the subsequent 2D-CNN forward pass. This ensures that raw temporal models and STFT-based models are compared under end-to-end inference conditions.

The inference procedure used batched prediction with a batch size of 128, consistent with the evaluation pipeline. Before recording inference time, several warm-up prediction runs were performed and excluded from the reported measurements to reduce the effect of initial graph tracing, GPU initialization, and runtime overhead. The inference-time measurement was then repeated on representative test batches, and the average inference time per segment was computed by dividing the total prediction time by the number of evaluated segments. The reported inference-time standard deviation was calculated across repeated prediction runs. Throughput was then calculated as the number of processed segments per second.

This analysis was included because a diagnostic model for induction motor condition monitoring should not only provide high classification performance, but should also be computationally efficient. In practical monitoring systems, especially edge-based or near-real-time applications, a compact model with low inference time may be preferable to a larger model with only marginally better accuracy. Therefore, the final model selection was based on the joint consideration of Macro-F1, worst-case Macro-F1, robustness score, parameter count, model size, and inference time.

It should be noted that the reported inference times were measured in a Google Colab NVIDIA Tesla T4 GPU environment using batched prediction. Therefore, these values should be interpreted as computational feasibility indicators rather than direct evidence of embedded-device deployment. Actual edge deployment requires additional evaluation on embedded or industrial hardware platforms, such as Raspberry Pi, Jetson Nano, industrial CPUs, or microcontroller-class TinyML devices.

### 3.10. Classical Machine-Learning Baseline Evaluation

To address the absence of non-deep-learning diagnostic baselines, classical machine-learning models were also evaluated in the revised study. Handcrafted features were extracted from the vibration signal and from the combined vibration–current signals. The extracted features included time-domain statistical descriptors, frequency-domain descriptors, FFT band-energy ratios, envelope-inspired features obtained using the Hilbert transform, and spectral-kurtosis-related descriptors. The envelope-inspired features were included to provide a simplified classical counterpart to envelope-analysis-based bearing diagnosis, while the spectral-kurtosis-related descriptors were included to capture impulsive spectral patterns associated with localized bearing faults.

For each signal channel, 33 handcrafted features were extracted. The vibration-only feature set therefore contained 33 features, while the vibration–current feature set contained 66 features. The evaluated classical classifiers included Random Forest, Extra Trees, Linear SVM, and XGBoost. These models were selected to provide representative tree-based, ensemble-based, margin-based, and boosting-based baselines.

The classical baselines were evaluated under two protocols. First, the measurement-wise split was used to compare baseline performance when all operating conditions were represented across the train and test sets. Second, the operating-condition holdout protocol was used to evaluate cross-condition generalization. The same Macro-F1, weighted F1, class-wise F1, worst-class F1, and robustness-score metrics were reported. This additional experiment was included to determine whether the CNN-based models provide advantages over conventional feature-engineering pipelines under operating-condition shift.

## 4. Results

### 4.1. Measurement-Wise Baseline Performance

The first experiment evaluated the raw 1D-CNN models under the measurement-wise validation protocol. In this setting, segments from the same measurement file were kept in the same split to avoid direct segment-level leakage, while all operating conditions were represented across the training, validation, and test sets. This experiment was used as a baseline before applying the stricter operating-condition holdout evaluation.

Table 5 presents the measurement-wise baseline results. The 1D-CNN Early Fusion model achieved the best performance, with an accuracy of 0.9259 and a Macro-F1 of 0.9251. The vibration-only model also performed strongly, reaching an accuracy of 0.8946 and a Macro-F1 of 0.8963. In contrast, the current-only model achieved substantially lower performance, with a Macro-F1 of 0.2594.

**Figure 4:**
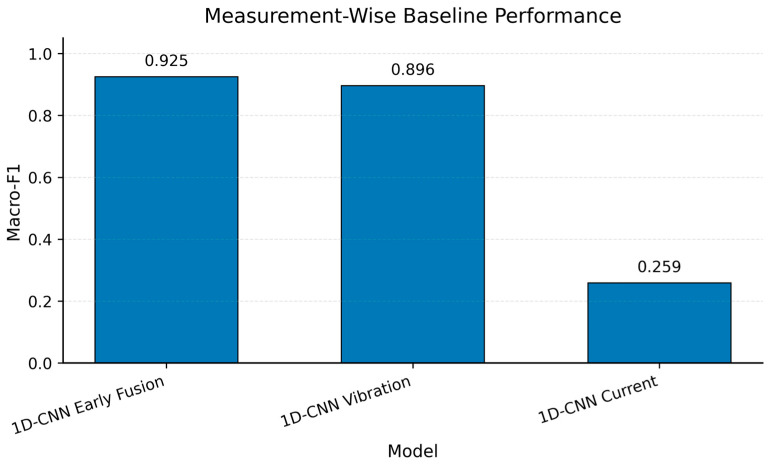
Measurement-wise baseline performance of the raw 1D-CNN models in terms of Macro-F1.

The measurement-wise Macro-F1 comparison of the raw 1D-CNN models is shown in Figure 4.

As shown in Figure 4, the 1D-CNN Early Fusion model achieved the highest measurement-wise Macro-F1, followed by the vibration-only model. The current-only model showed substantially weaker discrimination capability, indicating that one phase-current signal alone was not sufficient for reliable three-class diagnosis in this setting. Although the measurement-wise protocol provides a useful baseline, it does not fully evaluate robustness to unseen operating regimes. Therefore, the next experiment used strict operating-condition holdout validation.

### 4.2. Operating-Condition Holdout Performance

The condition-holdout experiment was conducted to evaluate cross-condition generalization. In each run, one operating condition was completely excluded from training and validation and used only for testing. This process was repeated for all four operating conditions so that each condition served once as the unseen test condition.

Table 6 reports the complete Macro-F1 results of all evaluated models across the four condition-holdout tests. The full condition-holdout results, including accuracy, loss, macro-precision, macro-recall, Macro-F1, weighted F1, and training time, are provided in Supplementary Table S1a. The corresponding Macro-F1 robustness summary is provided in Supplementary Table S1b.

As shown in Table 6, the performance of the evaluated models varied across unseen operating conditions. The condition N09_M07_F10 produced a clear performance degradation for most models and therefore represented the most challenging holdout condition. Under this condition, the 1D-CNN Early Fusion model achieved a Macro-F1 of 0.7474, outperforming the vibration-only 1D-CNN model and the STFT-based early fusion model. Although the Dual-Branch Feature Fusion model achieved the highest Macro-F1 under N09_M07_F10, its performance dropped considerably under N15_M01_F10, indicating lower stability across operating conditions.

The STFT-based models achieved strong performance under N15_M07_F04 and N15_M07_F10. However, their performance decreased substantially under N09_M07_F10. This suggests that time–frequency representations can be effective under some operating conditions but may be more sensitive to severe condition shifts.

The current-only model achieved consistently low Macro-F1 values across all holdout conditions. This confirms that the selected phase-current signal alone was not sufficient for reliable three-class diagnosis under unseen operating conditions. In contrast, vibration-based and vibration–current fusion models provided substantially stronger diagnostic performance.

**Figure 5:**
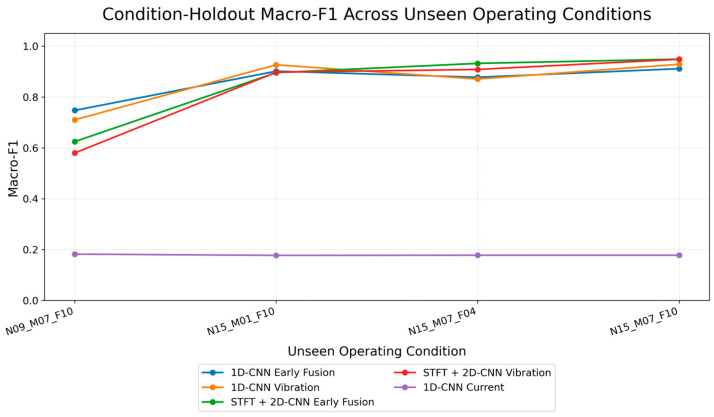
Condition-holdout Macro-F1 comparison across unseen operating conditions.

To further visualize the cross-condition behavior of the evaluated models, the condition-holdout Macro-F1 scores are shown in Figure 5.

Figure 5 confirms that N09_M07_F10 was the most difficult unseen operating condition for most models. The raw 1D-CNN Early Fusion model provided the best balance between average Macro-F1 and worst-case Macro-F1 among the lightweight models, while the current-only model failed to generalize reliably across all operating conditions.

### 4.3. Robustness Analysis Across Unseen Operating Conditions

To compare the models more systematically, robustness was evaluated using mean accuracy, standard deviation, worst-case accuracy, mean Macro-F1, standard deviation of Macro-F1, worst-case Macro-F1, and robustness score. The robustness score was defined as the mean Macro-F1 minus the standard deviation of Macro-F1 across the four condition-holdout tests.

Table 7 presents the robustness summary. The 1D-CNN Early Fusion model achieved the highest robustness score, with a mean Macro-F1 of 0.8595, a worst-case Macro-F1 of 0.7474, and a robustness score of 0.7834. The vibration-only 1D-CNN model was the second strongest model in terms of robustness, while the STFT-based models showed higher variability across conditions. More complex fusion mechanisms, including dual-branch feature fusion and gated fusion, did not outperform simple input-level early fusion.

**Figure 6:**
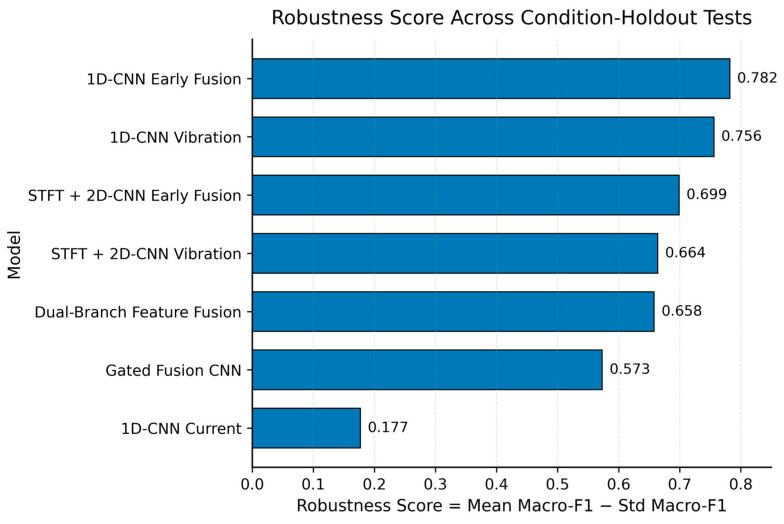
Robustness score comparison across condition-holdout tests.

The robustness-score comparison of the evaluated models is presented in Figure 6.

As shown in Figure 6, the 1D-CNN Early Fusion model achieved the highest robustness score among all evaluated approaches. This indicates that it provided the best balance between average Macro-F1 and stability across unseen operating conditions.

The results also show that architectural complexity did not guarantee better robustness. The gated fusion CNN introduced additional adaptive weighting, but its robustness score was lower than that of the simple early fusion model. This suggests that more flexible fusion mechanisms may be more sensitive to condition-specific patterns in the training data.

### 4.4. Robustness–Complexity Trade-Off

**Figure 7:**
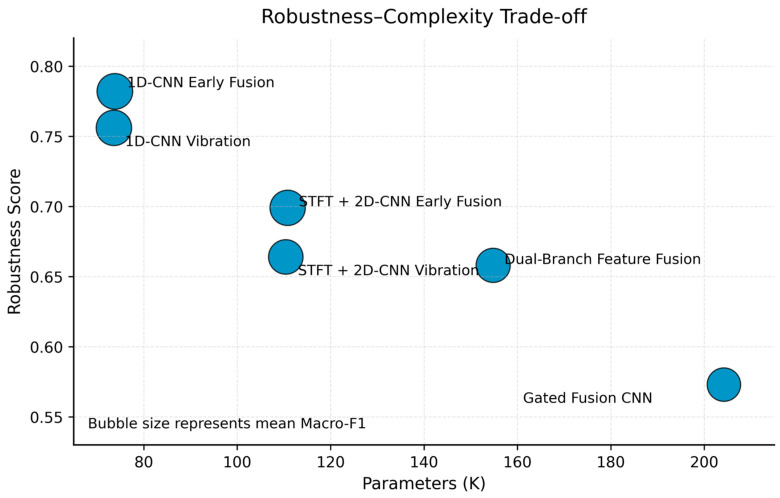
Robustness–complexity trade-off of the evaluated diagnostic models. Bubble size represents the mean Macro-F1 score.

To jointly evaluate diagnostic stability and architectural complexity, the robustness–complexity trade-off of the evaluated models is shown in Figure 7. This analysis compares each model in terms of parameter count and robustness score, while the bubble size represents the mean Macro-F1 score.

As shown in Figure 7, the 1D-CNN Early Fusion model achieved the highest robustness score while maintaining one of the lowest parameter counts. This indicates a favorable balance between cross-condition stability and model compactness. In contrast, the gated fusion CNN had a higher parameter count but produced a lower robustness score. Therefore, increasing fusion complexity did not necessarily improve cross-condition generalization.

This result is important for practical induction motor monitoring because diagnostic models should be selected not only by peak accuracy, but also by stability, computational cost, and worst-case behavior under unseen operating conditions.

### 4.5. Multi-Seed Validation of the Strongest Raw Temporal Models

Because the 1D-CNN Early Fusion and 1D-CNN Vibration models achieved the two highest robustness scores in the condition-holdout experiments, both models were further evaluated using multi-seed validation. Each model was trained and tested using three random seeds: 42, 123, and 2024. This experiment was conducted to verify whether the performance differences between the strongest raw temporal models remained stable across different random initializations.

Table 8 presents the multi-seed validation results. The vibration-only model achieved strong performance for some holdout conditions, especially N15_M01_F10. However, its performance varied substantially across random seeds and operating conditions. This instability was particularly evident for N09_M07_F10 and N15_M07_F04, where the standard deviation of Macro-F1 reached 0.2499 and 0.2604, respectively.

In contrast, the 1D-CNN Early Fusion model produced more stable results across all holdout conditions. For the most challenging condition, N09_M07_F10, the Early Fusion model achieved a Macro-F1 of 0.7378 ± 0.0293, while the vibration-only model achieved 0.6162 ± 0.2499. This result indicates that the addition of phase-current information improved the stability of the model under severe operating-condition shift.

Across all holdout conditions and random seeds, the 1D-CNN Vibration model achieved a mean Macro-F1 of 0.7652, a standard deviation of 0.2115, a worst-case Macro-F1 of 0.3279, and a robustness score of 0.5538. In comparison, the selected 1D-CNN Early Fusion model achieved a mean Macro-F1 of 0.8626, a standard deviation of 0.0776, a worst-case Macro-F1 of 0.7159, and a robustness score of 0.7850.

These results strengthen the model-selection decision. Although the vibration-only model was competitive in the single-run condition-holdout analysis, the multi-seed evaluation showed that it was less stable across random initializations. The 1D-CNN Early Fusion model provided a more reliable balance between average performance, worst-case performance, and cross-seed stability. Therefore, it was selected as the final diagnostic model.

**Figure 8:**
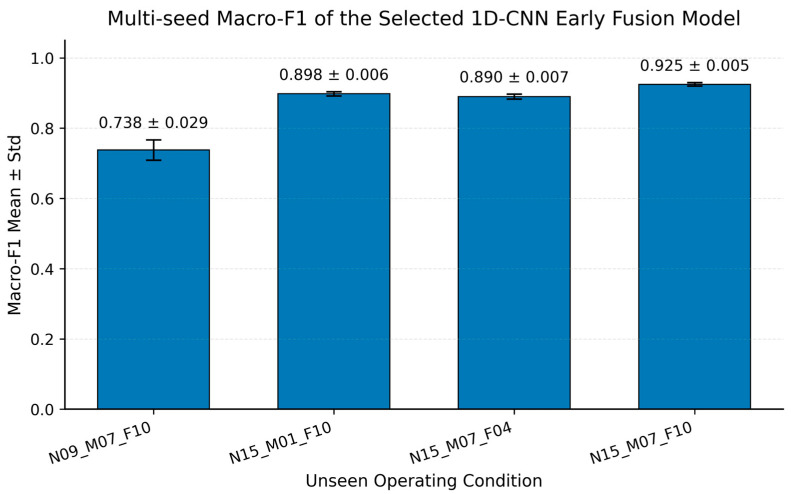
Multi-seed Macro-F1 performance of the selected 1D-CNN Early Fusion model across unseen operating conditions. Error bars indicate standard deviation across three random seeds.

After the comparative multi-seed analysis of the two strongest raw temporal models, the detailed Macro-F1 stability of the selected 1D-CNN Early Fusion model is visualized in Figure 8.

As shown in Figure 8, the selected model remained stable across random seeds for most operating conditions, while N09_M07_F10 remained the most challenging unseen condition with the lowest mean Macro-F1 and the highest variability. The multi-seed results support the conclusion that the selected model provides repeatable performance under operating-condition shift.

### 4.6. Complexity and Inference-Time Comparison

In addition to diagnostic performance, computational complexity was evaluated to assess practical applicability. Table 9 reports the number of parameters, model size, inference time per segment, and throughput for the evaluated models.

The selected 1D-CNN Early Fusion model required 73,891 parameters and had an estimated model size of 0.2819 MB. Its inference time was 0.5835 ms per segment, corresponding to a throughput of 1713.7462 segments per second. Although some models had lower measured inference time, they did not achieve the same robustness score. Therefore, the Early Fusion model provided the most favorable combination of robustness, compactness, and inference efficiency. Because the STFT operation was implemented as an internal TensorFlow layer in the STFT-based models, the reported inference times for these models include both time–frequency transformation and CNN forward propagation.

**Figure 9:**
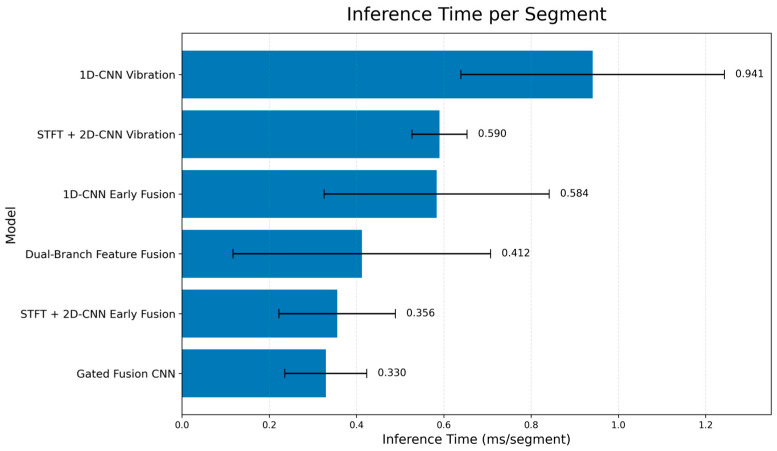
Inference-time comparison of the evaluated diagnostic models. Error bars indicate the standard deviation of inference time per segment.

The inference-time comparison of the evaluated diagnostic models is shown in Figure 9.

As shown in Figure 9, although some more complex models achieved lower inference time, the selected 1D-CNN Early Fusion model provided a better overall balance between robustness, compactness, and computational efficiency. Since inference time alone does not determine model suitability, these values should be interpreted together with robustness and worst-case performance.

Overall, among the evaluated CNN-based models, the results show that the 1D-CNN Early Fusion model achieved the most favorable robustness–complexity trade-off under operating-condition holdout validation. It improved over vibration-only learning in the most difficult condition, strongly outperformed current-only diagnosis, remained more robust than more complex fusion mechanisms, and maintained a small model size with low inference time.

### 4.7. Bearing-Code-Disjoint Validation

To further investigate whether the operating-condition holdout results were affected by bearing-identity sharing, the two strongest raw temporal models were evaluated under the additional bearing-code-disjoint validation protocol. In this experiment, the test bearing codes were completely unseen during training and validation. Therefore, this setting was substantially stricter than the operating-condition holdout protocol.

Table 10 summarizes the bearing-code-disjoint validation results. Both models showed a substantial performance reduction compared with the operating-condition holdout results, confirming that generalization to unseen bearing identities is more challenging than generalization to unseen operating conditions alone. The 1D-CNN Vibration model achieved a mean Macro-F1 of 0.5616 ± 0.2500, while the 1D-CNN Early Fusion model achieved a mean Macro-F1 of 0.5485 ± 0.2443. The corresponding robustness scores were 0.3116 and 0.3042, respectively.

A paired statistical comparison was also performed across the 40 paired runs. As shown in Table 11, the mean paired Macro-F1 difference between the 1D-CNN Early Fusion and 1D-CNN Vibration models was −0.0130. The Wilcoxon signed-rank test did not indicate a statistically significant difference between the two models under the bearing-code-disjoint setting (*p* = 0.2016). The bootstrap 95% confidence interval for the mean paired difference was [−0.0336, 0.0069], which also included zero.

These results indicate that the advantage of early vibration–current fusion observed under operating-condition holdout does not directly transfer to the stricter bearing-code-disjoint setting. Therefore, the main robustness conclusion of this study should be interpreted specifically in the context of operating-condition shift. Complete generalization to unseen bearing identities remains more difficult and requires further investigation using larger multi-bearing and cross-dataset validation protocols.

### 4.8. Classical Machine-Learning Baseline Performance

Classical machine-learning baselines were evaluated to compare the CNN-based models with conventional handcrafted-feature-based diagnostic pipelines. The measurement-wise results are summarized in Table 12. Under measurement-wise validation, the classical baselines achieved very high performance, especially when vibration and current features were combined. The strongest measurement-wise baseline was XGBoost using vibration–current features, with a mean accuracy of 0.9935 and a mean Macro-F1 of 0.9937.

Although these measurement-wise results are high, they should be interpreted with caution because all operating conditions are represented across the train and test sets. Therefore, the classical baselines were also evaluated under the stricter operating-condition holdout protocol.

Table 13 reports the condition-holdout performance of the classical baselines. Under this stricter setting, the best-performing baseline was XGBoost using vibration features, achieving a mean Macro-F1 of 0.8582 and a robustness score of 0.7040. The vibration–current XGBoost baseline achieved a similar mean Macro-F1 of 0.8514 but showed higher variability, resulting in a lower robustness score of 0.6726. These results indicate that handcrafted vibration features combined with a strong tree-based boosting classifier can provide competitive cross-condition robustness.

The classical baseline results provide two important observations. First, very high measurement-wise performance can be achieved even with handcrafted features, confirming that measurement-wise validation alone may overestimate deployment-oriented diagnostic capability. Second, under condition-holdout validation, the strongest classical baseline was competitive with the CNN-based models. However, the selected 1D-CNN Early Fusion model still provided a favorable balance of raw-signal learning, compact model size, and robustness-oriented performance without requiring manual feature extraction. These results strengthen the comparative scope of the study and show that the proposed evaluation framework is not limited to deep learning models alone. Importantly, these results also indicate that the CNN-based early-fusion model should not be interpreted as universally superior to classical feature-based learning. Instead, it provides a compact raw-signal learning alternative with competitive robustness, while XGBoost with handcrafted vibration features remains a strong classical baseline.

## 5. Discussion

### 5.1. Influence of Operating-Condition Shift

The results confirm that operating-condition shift substantially affects induction motor bearing fault diagnosis. Under measurement-wise validation, the 1D-CNN Early Fusion model achieved a Macro-F1 of 0.9251, indicating that the model could learn discriminative fault-related patterns when all operating conditions were represented across the data splits. However, under strict condition-holdout validation, the performance of all models became more condition-dependent. This demonstrates that measurement-wise performance alone is not sufficient for assessing practical diagnostic reliability.

The most challenging unseen condition was N09_M07_F10. Most models showed a clear performance reduction under this condition compared with the other three holdout settings. This suggests that the signal distribution of N09_M07_F10 differs more strongly from the remaining operating conditions. A likely physical reason is that N09_M07_F10 corresponds to the lower rotational-speed regime compared with the N15 conditions, while also involving a different combination of load and radial force. Therefore, the fault-related vibration and current signatures may shift more strongly relative to the training conditions, making this holdout case more difficult.

This behavior is consistent with the operating-condition shift challenge discussed in Section 2.2 and Section 3.4.2, where changes in speed, load, radial force, or working-condition distributions were identified as important factors affecting diagnostic generalization. Therefore, the condition-holdout protocol used in this study provides a stricter and more deployment-oriented evaluation than conventional measurement-wise splitting.

### 5.2. Contribution of Vibration and Phase-Current Signals

The single-modality results show that vibration is the dominant diagnostic source in the tested setup. The 1D-CNN Vibration model achieved strong Macro-F1 values in several holdout conditions, which is expected because bearing defects directly affect mechanical vibration patterns. This result is consistent with the role of vibration-based diagnosis discussed in the earlier literature review, where vibration signals were shown to provide direct mechanical information about bearing degradation.

In contrast, the current-only model produced consistently weak Macro-F1 values across all holdout conditions. This does not mean that current signals are irrelevant for bearing fault diagnosis. As discussed in Section 2.1 and Section 3.3, phase-current measurements can contain useful complementary information for induction motor monitoring. However, in the present three-class Paderborn subset, one phase-current channel alone was not sufficient to reliably distinguish healthy, artificial-damage, and real-damage states under unseen operating conditions.

The key finding is that phase current became useful when combined with vibration. The Early Fusion model achieved a higher worst-case Macro-F1 and a higher robustness score than the vibration-only model. This indicates that the current signal provided complementary information that improved robustness, even though it was not reliable as a standalone modality. Therefore, in this framework, phase current should be interpreted as an auxiliary modality that supports vibration-based diagnosis rather than replacing vibration sensing.

From an electromechanical perspective, this complementary behavior can be explained by the different physical pathways through which bearing faults appear in vibration and current measurements. Localized bearing defects primarily generate impulsive mechanical responses, which are directly reflected in vibration signals. At the same time, these mechanical disturbances may slightly modulate the electromagnetic behavior of the induction motor through air-gap variation, load-torque fluctuation, rotor–stator interaction, and electromechanical coupling. As a result, the phase-current signal may contain weak but useful auxiliary information related to the motor response under changing operating conditions.

The early-fusion architecture combines vibration and phase-current segments at the input level, allowing the first convolutional filters to learn joint local temporal patterns from both modalities before higher-level abstraction. This may be particularly useful under operating-condition shift, where vibration amplitudes and spectral components can change with speed, load, and radial force. In this setting, the current channel may help stabilize the learned representation by providing indirect information about the electromechanical operating state. Therefore, the observed advantage of early fusion should be interpreted as a physically plausible complementarity between direct mechanical vibration signatures and indirect electrical current signatures, rather than as a purely empirical improvement.

The consistently weak current-only results should not be interpreted as evidence that motor current signals are generally uninformative for bearing fault diagnosis. Rather, they indicate that the evaluated single-channel raw-current pipeline was insufficient for reliable standalone three-class diagnosis under unseen operating conditions. Several factors may explain this behavior. First, only one phase-current channel was used, whereas multi-phase current analysis may better capture electrical asymmetries and fault-related modulation patterns. Second, the current signal was processed using the same raw temporal CNN structure as the vibration signal, without current-specific preprocessing such as harmonic analysis, sideband analysis, demodulation, or filtering around characteristic electrical components. Third, bearing faults primarily generate mechanical impulses, and their effects on current may be weak, indirect, and easily masked by load-dependent components, supply-related variations, and operating-condition changes. Fourth, the synchronization and phase relationship between vibration and current measurements were not explicitly optimized.

Therefore, the current-only results mainly show the limitation of the specific raw single-phase-current pipeline used in this study. They do not rule out the diagnostic value of current signals when more appropriate current-oriented preprocessing, multi-phase acquisition, harmonic or sideband features, or current-specific model architectures are used. Future work should therefore investigate multi-phase current inputs, motor-current signature analysis, current-domain filtering, phase-alignment strategies, and hybrid vibration–current representations to more fully assess the standalone and auxiliary diagnostic value of current signals.

### 5.3. Early Fusion Versus More Complex Fusion Strategies

A central result of this study is that the simple 1D-CNN Early Fusion model provided the best robustness–complexity trade-off. More complex fusion strategies, including Dual-Branch Feature Fusion and Gated Fusion CNN, did not improve overall robustness. This suggests that additional fusion complexity can increase sensitivity to condition-specific patterns learned from the training conditions.

Recent multi-sensor bearing fault diagnosis studies have shown that advanced fusion strategies, such as double-level data fusion, multichannel sample fusion, and feature fusion, can improve fault representation under variable or time-varying operating conditions [45]. Similarly, multi-branch feature cross-fusion models have been proposed to improve multi-sensor feature integration and diagnostic discrimination [46]. However, the present results indicate that such additional fusion complexity does not automatically guarantee better robustness when the validation protocol is based on strict unseen operating-condition testing.

One possible explanation is that feature-level and gated fusion architectures learn modality-specific representations before combining them. While this can be beneficial when both modalities contain stable and complementary information, it may also increase the risk of learning condition-dependent features. In contrast, input-level early fusion allows the convolutional filters to learn joint local temporal patterns directly from the two-channel signal. This simpler representation may be less prone to overfitting under strict condition-holdout validation.

This finding is important because multimodal and multi-source fault diagnosis has received increasing attention, but fusion strategies should not be evaluated only by average accuracy or peak performance. Instead, they should be compared using worst-case behavior, cross-condition variability, random-seed stability, parameter count, and inference efficiency. From this perspective, the selected 1D-CNN Early Fusion model offers a more practical solution than the evaluated feature-level and gated fusion alternatives.

Although this study did not perform a separate block-level ablation for every architectural component, the evaluated model set provides a modality-level and fusion-strategy-level ablation. Specifically, vibration-only, current-only, early-fusion, STFT-based, dual-branch, and gated-fusion variants were compared under the same preprocessing and validation settings.

### 5.4. Raw Temporal Learning Versus STFT-Based Learning

The STFT-based 2D-CNN models achieved strong performance under some holdout conditions, especially N15_M07_F04 and N15_M07_F10. This indicates that time–frequency representations can make fault-related spectral patterns more visible under certain operating regimes. Recent studies have also shown that time–frequency transformations such as STFT, CWT, Hilbert–Huang transform, and Wigner–Ville distribution can provide informative two-dimensional representations for bearing and rotating machinery fault diagnosis [47]. Time–frequency dual-domain and feature-fusion models have also been proposed to improve fault feature extraction under noisy or sample-limited conditions [48].

However, the STFT-based models showed larger performance degradation under N09_M07_F10. This suggests that time–frequency representations may emphasize spectral structures that are not equally stable across all operating conditions. In contrast, raw 1D temporal learning avoids explicit transformation and preserves waveform-level information, which may be advantageous when spectral characteristics shift between training and testing conditions.

Recent multiscale time–frequency deep learning studies have reported strong performance under complex and variable operating conditions, but they also rely on more elaborate feature-extraction mechanisms and model structures [49]. Therefore, the results of the present study should not be interpreted as evidence that STFT-based learning is ineffective. Rather, they show that its advantage depends on the operating condition, input modality, and validation protocol. Under strict condition-holdout evaluation, raw temporal early fusion provided a more stable robustness–complexity trade-off than the evaluated STFT-based alternatives.

The STFT configuration used in this study was intentionally kept fixed across all conditions and modalities to ensure a controlled comparison. However, STFT-based diagnosis can be sensitive to window length, hop size, frequency resolution, and the relationship between the transform parameters and bearing characteristic frequencies such as BPFO, BPFI, BSF, and FTF. Therefore, the STFT results should be interpreted as representative of the evaluated configuration rather than as an exhaustive assessment of all possible time–frequency settings. Future work should include STFT parameter sensitivity analysis and explicit alignment with bearing characteristic frequencies.

### 5.5. Multi-Seed Stability and Model Selection

The multi-seed evaluation strengthened the final model-selection decision. In the single-run condition-holdout analysis, the 1D-CNN Vibration and 1D-CNN Early Fusion models achieved comparable mean Macro-F1 values. However, the multi-seed comparison showed that the vibration-only model was considerably less stable across random initializations and validation-split variations.

Across all holdout conditions and random seeds, the 1D-CNN Vibration model achieved a mean Macro-F1 of 0.7652, a standard deviation of 0.2115, a worst-case Macro-F1 of 0.3279, and a robustness score of 0.5538. In comparison, the 1D-CNN Early Fusion model achieved a mean Macro-F1 of 0.8626, a standard deviation of 0.0776, a worst-case Macro-F1 of 0.7159, and a robustness score of 0.7850. These results indicate that early fusion improved not only average diagnostic performance, but also worst-case robustness and cross-seed stability.

The improvement was particularly important under N09_M07_F10, which was the most challenging unseen operating condition. Under this condition, the Early Fusion model achieved a Macro-F1 of 0.7378 ± 0.0293, while the vibration-only model achieved 0.6162 ± 0.2499. This large difference in variability suggests that adding phase-current information helped stabilize the diagnostic decision boundary when the operating condition shifted severely.

Therefore, the selected 1D-CNN Early Fusion model was not chosen only because of its peak performance. It was selected because it provided the most reliable combination of mean Macro-F1, worst-case Macro-F1, robustness score, cross-seed stability, compactness, and inference efficiency. This supports the main argument of the study that deployment-oriented model selection should consider stability and robustness, not only single-run accuracy.

### 5.6. Bearing-Identity Generalization and Classical Baselines

The additional bearing-code-disjoint validation provides a stricter perspective on the robustness claims of this study. While the operating-condition holdout protocol evaluates generalization to unseen speed/load/radial-force conditions, it may still include the same bearing identities under other operating conditions in the training set. The bearing-code-disjoint experiment removed this overlap by excluding complete bearing codes from training and validation.

The results showed that both raw temporal models experienced a substantial performance reduction under bearing-code-disjoint testing. The 1D-CNN Vibration model achieved a mean Macro-F1 of 0.5616, while the 1D-CNN Early Fusion model achieved 0.5485. The paired Wilcoxon test did not show a statistically significant advantage for early fusion under this stricter setting. This finding indicates that the early-fusion advantage observed under operating-condition shift should not be interpreted as complete bearing-identity generalization. Instead, the main claim of the paper should be limited to robustness under operating-condition shift.

The classical baseline experiment also provides an important comparison. Handcrafted vibration features combined with XGBoost achieved a mean Macro-F1 of 0.8582 under condition-holdout validation, which is competitive with the CNN-based results. This confirms that classical diagnostic pipelines remain strong baselines when informative time-domain, frequency-domain, envelope-inspired, and spectral-kurtosis features are available. However, such methods require explicit feature engineering, while the CNN-based models learn directly from raw signal segments. Therefore, the results do not show that deep learning is universally superior; rather, they show that lightweight raw temporal CNNs and strong classical feature-based baselines can both be effective under operating-condition shift.

### 5.7. Practical Implications

The proposed evaluation framework has several practical implications for induction motor condition monitoring and IIoT-oriented predictive maintenance. First, deployment-oriented validation is essential. A model that performs well under measurement-wise validation may still show reduced reliability under unseen operating conditions. Therefore, condition-holdout validation should be considered when evaluating diagnostic models intended for real industrial monitoring scenarios.

Second, vibration remains the most informative single modality in the tested setup, but phase current can improve robustness when used as an auxiliary modality. This is practically useful because current measurements are often already available in motor drive systems. Combining current with vibration may therefore improve diagnostic robustness without requiring a highly complex fusion architecture.

Third, the selected 1D-CNN Early Fusion model is lightweight and computationally efficient. It required only 73,891 parameters, had an estimated size of 0.2819 MB, and achieved an inference time of 0.5835 ms per segment in the tested Google Colab Tesla T4 environment. These results indicate computational feasibility and low model complexity, which are desirable properties for edge-oriented condition monitoring. However, they should not be interpreted as direct proof of embedded-device deployment. Actual edge deployment requires additional validation on embedded or industrial hardware platforms, where latency, memory footprint, energy consumption, and preprocessing overhead may differ from the Colab GPU environment [50].

Finally, the results support the broader direction of IIoT-based predictive maintenance systems, where sensor data, diagnostic models, and deployment infrastructure must be considered together. Edge-cloud predictive maintenance frameworks have shown that real-time data acquisition, predictive modeling, and deployment architecture are central requirements for practical smart manufacturing systems [51]. From this perspective, the proposed early-fusion model can be considered a computationally lightweight candidate for near-real-time induction motor monitoring, but its actual edge-deployment suitability must be verified through dedicated experiments on embedded or industrial monitoring hardware.

### 5.8. Limitations and Future Work

Several limitations should be considered. First, the experiments were conducted on a controlled medium subset of the Paderborn bearing dataset. Although this dataset is suitable for analyzing operating-condition shift using vibration and current signals, future studies should validate the proposed framework on additional bearing datasets and real industrial motor systems. Cross-dataset validation would provide a stronger assessment of whether the selected early-fusion strategy can generalize beyond the specific experimental structure used in this study.

Second, the robustness score used in this study has an important limitation. Although this score is useful as a compact descriptive indicator of cross-condition stability, its formulation is heuristic and should not be interpreted as a formal theoretical generalization bound. The score combines mean Macro-F1 and cross-condition variability, but it cannot replace detailed reporting of class-wise F1, worst-case Macro-F1, confidence intervals, statistical testing, or model-complexity measures. For this reason, the revised analysis interprets the robustness score only as one component of the model-selection process, together with Macro-F1, worst-case performance, cross-seed stability, parameter count, inference time, and paired statistical testing.

Third, although the experiments in this study were conducted on induction motor bearing data, the proposed evaluation framework can be conceptually extended to other rotating electrical machines and electromechanical systems, such as permanent magnet synchronous motors, brushless DC motors, gearboxes, pumps, and motor-driven industrial machinery. The general evaluation idea—comparing raw temporal learning, time–frequency representations, multimodal fusion strategies, classical feature-based baselines, and strict domain-holdout validation—is not specific to induction motors. However, the numerical results reported in this study should not be directly generalized to other machine types without additional validation.

Different machines may exhibit different fault mechanisms, electromagnetic coupling behavior, sensor configurations, control strategies, speed/load profiles, and dominant fault-frequency characteristics. For example, permanent magnet synchronous motors and brushless DC motors may produce different current signatures because of their magnetic structure and inverter-control behavior, while gearboxes and pumps may exhibit different vibration transfer paths and mechanical fault-frequency patterns. Therefore, applying the proposed framework to other machines would require machine-specific signal interpretation, appropriate operating-condition definitions, and independent validation datasets. Future work should extend the framework to other electromechanical systems to evaluate whether the observed robustness–complexity trends remain consistent.

Fourth, this study used one vibration signal and one phase-current signal. Future work may examine whether multiple current phases, multiple vibration channels, acoustic signals, thermal measurements, or other sensor modalities can further improve robustness. In addition, more advanced sensor-fusion strategies could be evaluated, provided that their added complexity is justified by improved worst-case robustness and deployment efficiency.

Fifth, the study focused on offline segmented diagnosis. Practical deployment may involve online streaming, variable window lengths, missing data, sensor noise, and real-time decision logic. Therefore, future work should evaluate the selected model in online or edge-based monitoring scenarios. Recent edge-deployable TinyML studies have shown that bearing fault diagnosis models can be implemented on resource-constrained microcontrollers, but such deployment requires careful evaluation of latency, memory footprint, energy consumption, and communication requirements [52].

Sixth, although the revised study added a paired Wilcoxon signed-rank test and bootstrap confidence interval for the bearing-code-disjoint experiment, the main multi-seed condition-holdout comparison was still limited to the two strongest raw temporal models. Future studies should extend formal statistical testing to all evaluated CNN architectures and include a larger number of random seeds.

Seventh, the revised study added a bearing-code-disjoint validation experiment to evaluate unseen bearing-identity generalization. However, this experiment was conducted on the same controlled Paderborn subset and only for the two strongest raw temporal models. Future work should evaluate combined bearing-code and operating-condition holdout protocols, cross-dataset validation, and larger multi-bearing experimental settings.

Finally, although several CNN-based architectures were compared, other approaches such as domain generalization, adaptive normalization, lightweight attention mechanisms, transformer-based models, and transfer learning could be evaluated under the same condition-holdout framework. Recent domain generalization studies for bearing fault diagnosis under unknown operating conditions indicate that learning condition-invariant or decoupled fault representations is a promising direction for improving practical diagnostic robustness [53]. Such comparisons would further clarify which strategies are most effective for robust motor bearing diagnosis under severe operating-condition shifts.

### 5.9. Summary of Discussion

Overall, the results show that strict condition-holdout validation provides a more realistic assessment of induction motor bearing fault diagnosis than measurement-wise validation alone. Among the evaluated CNN models, the selected 1D-CNN Early Fusion model achieved the most favorable balance between mean performance, worst-case Macro-F1, cross-seed stability, model compactness, and computational feasibility.

However, the additional bearing-code-disjoint validation shows that robustness under operating-condition shift should not be interpreted as complete generalization to unseen bearing identities. The classical baseline results further show that handcrafted vibration features combined with XGBoost remain highly competitive under condition-holdout validation. Therefore, practical diagnostic systems should be evaluated using multiple complementary criteria, including validation protocol strictness, worst-case behavior, statistical stability, model complexity, and the availability or cost of feature engineering.

## 6. Conclusions

This study presented a robustness-oriented comparative framework for induction motor bearing fault diagnosis under operating-condition shift using vibration and phase-current signals. Unlike conventional measurement-wise evaluation, the proposed framework emphasized strict condition-holdout validation, where one operating condition was completely excluded from training and used only for testing. This protocol provided a more realistic assessment of cross-condition generalization and practical diagnostic robustness.

Several CNN-based diagnostic strategies were evaluated, including raw 1D-CNN Vibration learning, current-only learning, 1D-CNN Vibration–current early fusion, STFT-based 2D-CNN representations, dual-branch feature fusion, and gated fusion. The results showed that current-only diagnosis was not sufficient for reliable three-class bearing fault classification under unseen operating conditions. Vibration-based learning provided strong diagnostic performance, while vibration–current early fusion achieved the best overall robustness–complexity trade-off.

The multi-seed comparison further strengthened the model-selection decision. Although the vibration-only 1D-CNN achieved competitive performance in the single-run condition-holdout analysis, it showed substantially higher variability across random seeds. Across all holdout conditions and random seeds, the vibration-only model achieved a mean Macro-F1 of 0.7652, a worst-case Macro-F1 of 0.3279, and a robustness score of 0.5538. In contrast, the selected 1D-CNN Early Fusion model achieved a mean Macro-F1 of 0.8626, a worst-case Macro-F1 of 0.7159, and a robustness score of 0.7850. These results indicate that adding phase-current information improved not only average performance but also worst-case robustness and cross-seed stability.

The selected 1D-CNN Early Fusion model remained computationally lightweight, requiring only 73,891 parameters, with an estimated model size of 0.2819 MB and an inference time of 0.5835 ms per segment in the tested Colab GPU environment. These results support computational feasibility, but embedded-device deployment remains to be validated on dedicated edge or industrial monitoring hardware.

These findings suggest that, among the evaluated CNN architectures, simple input-level fusion of vibration and phase-current signals can be more practical than more complex fusion mechanisms when operating-condition shift, worst-case performance, model size, and inference efficiency are considered together.

Overall, the study demonstrates that diagnostic models for induction motor condition monitoring should not be evaluated only by peak classification accuracy. Instead, measurement-wise performance, condition-holdout robustness, worst-case Macro-F1, cross-seed stability, computational complexity, and inference time should all be considered. From this perspective, the proposed 1D-CNN Early Fusion approach provides a lightweight and effective CNN-based solution for bearing fault diagnosis under unseen operating conditions, while the added classical baseline results show that feature-based XGBoost remains a strong alternative.

The additional bearing-code-disjoint validation showed that complete generalization to unseen bearing identities is more challenging than operating-condition generalization alone. Under this stricter setting, the vibration-only 1D-CNN achieved a mean Macro-F1 of 0.5616, while the 1D-CNN Early Fusion model achieved 0.5485. The paired statistical test did not show a significant advantage for early fusion. Therefore, the main conclusion of this study is limited to operating-condition shift rather than complete bearing-identity generalization.

The added classical machine-learning baselines also showed that handcrafted vibration features combined with XGBoost remain highly competitive under condition-holdout validation, achieving a mean Macro-F1 of 0.8582. This confirms the importance of comparing deep learning models with strong classical diagnostic baselines. Overall, the study supports the use of deployment-oriented evaluation criteria rather than peak accuracy alone, while also showing that both lightweight raw temporal CNNs and classical feature-based models can provide useful diagnostic robustness under operating-condition shift.

Future work may extend this framework to additional bearing datasets, real industrial motor environments, multi-sensor acquisition systems, online streaming diagnosis, and edge-based deployment. Further investigation of domain generalization, adaptive normalization, transfer learning, and lightweight attention-based models may also improve robustness under more severe operating-condition variations. The present numerical findings are specific to the evaluated induction-motor Paderborn subset, and extension to other electrical machines or rotating systems requires independent machine-specific validation. Future work should also include STFT parameter sensitivity analysis, explicit bearing-characteristic-frequency-informed time–frequency features, combined bearing-code and operating-condition holdout testing, and embedded-device latency and memory evaluation.

## Figures and Tables

**Table 1:** Comparison of representative bearing fault diagnosis studies and the present work.

Study	Signal Modality	Method	Validation Focus	Main Limitation
Zhang et al. [6]	Vibration	Deep CNN with improved training strategy	Load/noise-varying diagnosis	Single-modality vibration focus; no vibration–current fusion
Eren et al. [7]	Vibration	Compact adaptive 1D-CNN	Raw temporal CNN classification	No explicit operating-condition holdout or multimodal analysis
Hoang and Kang [9]	Vibration	CNN with vibration-image representation	Image-based vibration diagnosis	Time–frequency/image representation only; no current modality
Islam and Kim [10]	Vibration	Wavelet packet transform + deep CNN	2D transformed-signal classification	Additional preprocessing; no vibration–current fusion
Barcelos and Cardoso [14]	Stator current	Current-based deep learning diagnosis	Current-based motor monitoring	Current-only focus; no direct comparison with vibration fusion
Dong et al. [17]	Multi-source data	Lightweight 1D-CNN	Multi-source fault diagnosis	Limited comparison of fusion strategies and robustness metrics
Alam et al. [19]	Vibration + motor phase current	1D-CNN with transfer learning	Variable-condition and transfer-learning evaluation	Transfer-learning-oriented; no direct comparison of raw 1D, STFT 2D, and fusion variants
Ayankoso et al. [20]	Vibration + current	Deep learning and machine-learning models	Modality comparison	Does not focus on robustness score and inference-complexity trade-off
Wang et al. [37]	Vibro-acoustic data	1D-CNN fusion	Multi-sensor fusion	Vibro-acoustic rather than vibration–current fusion
This study	Vibration + phase current	Raw 1D-CNN, STFT + 2D-CNN, early fusion, dual-branch fusion, gated fusion, and classical feature-based baselines	Measurement-wise, operating-condition holdout, multi-seed, bearing-code-disjoint validation, paired statistical testing, classical baseline comparison, and inference analysis	Focused on a controlled Paderborn subset; embedded-device validation, STFT parameter sensitivity analysis, combined bearing-code/condition holdout, and cross-dataset testing remain future work

**Table 2:** Summary of the medium Paderborn subset used in this study.

Property	Description
Dataset	Paderborn bearing dataset
Bearing codes	22
Classes	Healthy, artificial damage, real damage
Healthy bearing codes	6
Artificial-damage bearing codes	8
Real-damage bearing codes	8
Operating conditions	4
Signal modalities	Vibration and phase current
Window size	4096 samples
Segments per measurement file	10
Total segments	17,590
Measurement files	1759
Excluded files	1 artificial-damage file
Excluded file name	N15_M01_F10_KA08_2.mat
Validation settings	Measurement-wise split, operating-condition holdout, bearing-code-disjoint validation, and classical baseline evaluation

**Table 3:** Operating-condition holdout validation protocol.

Holdout Test Condition	Training Conditions
N09_M07_F10	N15_M01_F10, N15_M07_F04, N15_M07_F10
N15_M01_F10	N09_M07_F10, N15_M07_F04, N15_M07_F10
N15_M07_F04	N09_M07_F10, N15_M01_F10, N15_M07_F10
N15_M07_F10	N09_M07_F10, N15_M01_F10, N15_M07_F04

**Table 4:** Summary of the evaluated CNN-based diagnostic architectures.

Model	Input Modality	Input Representation	Architecture Summary	Fusion Strategy	Trainable Parameters
1D-CNN Vibration	Vibration	(4096\times 1) raw signal	Three Conv1D blocks with 32/64/128 filters, kernel sizes 9/7/5, same padding, batch normalization, ReLU, max-pooling, global average pooling, dense layer with 128 units, dropout 0.30, and softmax output	None	73,603
1D-CNN Current	Phase current	(4096\times 1) raw signal	Same raw 1D-CNN backbone as the vibration-only model	None	73,603
1D-CNN Early Fusion	Vibration + phase current	(4096\times 2) raw two-channel signal	Same raw 1D-CNN backbone with vibration and phase-current signals stacked as two input channels	Input-level fusion	73,891
STFT + 2D-CNN Vibration	Vibration	Single-channel log-magnitude STFT map	Three Conv2D blocks with 32/64/128 filters, (3\times 3) kernels, same padding, batch normalization, ReLU, max-pooling, global pooling/classification head, and softmax output	None	110,467
STFT + 2D-CNN Early Fusion	Vibration + phase current	Two-channel log-magnitude STFT map	Same 2D-CNN backbone with vibration and phase-current STFT maps stacked as two channels	Input-level time–frequency fusion	110,755
Dual-Branch Feature Fusion	Vibration + phase current	Two separate raw (4096\times 1) inputs	Two parallel 1D-CNN feature extractors followed by feature concatenation and softmax classification	Feature-level fusion	154,755
Gated Fusion CNN	Vibration + phase current	Two separate raw (4096\times 1) inputs	Two parallel 1D-CNN branches with learnable modality gating before final classification	Adaptive gated fusion	204,035

**Table 5:** Measurement-wise baseline performance of raw 1D-CNN models.

Model	Accuracy	Macro-Precision	Macro-Recall	Macro-F1	Weighted F1	Training Time (s)
1D-CNN Early Fusion	0.925852	0.936199	0.919306	0.925063	0.925414	44.524036
1D-CNN Vibration	0.894602	0.905471	0.892687	0.896281	0.894767	34.646318
1D-CNN Current	0.360227	0.256767	0.348851	0.259403	0.276565	24.899763

**Table 6:** Complete condition-holdout Macro-F1 results across unseen operating conditions.

Model	N09_M07_F10	N15_M01_F10	N15_M07_F04	N15_M07_F10	Mean Macro-F1	Worst Macro-F1
1D-CNN Vibration	0.7103	0.9266	0.8706	0.9288	0.8591	0.7103
1D-CNN Current	0.1821	0.1773	0.1778	0.1778	0.1787	0.1773
1D-CNN Early Fusion	0.7474	0.9016	0.8777	0.9115	0.8595	0.7474
STFT + 2D-CNN Vibration	0.5799	0.8973	0.9085	0.9483	0.8335	0.5799
STFT + 2D-CNN Early Fusion	0.6246	0.8958	0.9323	0.9490	0.8504	0.6246
Dual-Branch Feature Fusion	0.7756	0.6093	0.8863	0.9034	0.7937	0.6093
Gated Fusion CNN	0.7012	0.5215	0.8586	0.9046	0.7465	0.5215

**Table 7:** Robustness summary across operating-condition holdout tests.

Model	Mean Accuracy	Std Accuracy	Worst Accuracy	Mean Macro-F1	Std Macro-F1	Worst Macro-F1	Robustness Score
1D-CNN Early Fusion	0.8568	0.0850	0.7316	0.8595	0.0761	0.7474	0.7834
1D-CNN Vibration	0.8564	0.1114	0.6948	0.8591	0.1028	0.7103	0.7563
STFT + 2D-CNN Early Fusion	0.8543	0.1505	0.6311	0.8504	0.1522	0.6246	0.6983
STFT + 2D-CNN Vibration	0.8349	0.1712	0.5805	0.8335	0.1705	0.5799	0.6630
Dual-Branch Feature Fusion	0.8195	0.0900	0.7237	0.7937	0.1353	0.6093	0.6584
Gated Fusion CNN	0.7573	0.1577	0.5640	0.7465	0.1734	0.5215	0.5731
1D-CNN Current	0.3637	0.0013	0.3622	0.1787	0.0023	0.1773	0.1765

**Table 8:** Multi-seed validation comparison of the 1D-CNN Vibration and 1D-CNN Early Fusion models.

Model	Test Condition	Accuracy Mean	Accuracy Std	Macro-F1 Mean	Macro-F1 Std	Weighted F1 Mean	Weighted F1 Std
1D-CNN Vibration	N09_M07_F10	0.6350	0.1960	0.6162	0.2499	0.6035	0.2542
1D-CNN Vibration	N15_M01_F10	0.9051	0.0184	0.9048	0.0169	0.9048	0.0184
1D-CNN Vibration	N15_M07_F04	0.7420	0.2609	0.7409	0.2604	0.7425	0.2595
1D-CNN Vibration	N15_M07_F10	0.8048	0.2171	0.7991	0.2242	0.8027	0.2210
1D-CNN Early Fusion	N09_M07_F10	0.7222	0.0330	0.7378	0.0293	0.7256	0.0326
1D-CNN Early Fusion	N15_M01_F10	0.8986	0.0074	0.8983	0.0063	0.8981	0.0073
1D-CNN Early Fusion	N15_M07_F04	0.8905	0.0077	0.8896	0.0075	0.8903	0.0079
1D-CNN Early Fusion	N15_M07_F10	0.9269	0.0055	0.9248	0.0047	0.9271	0.0052

**Table 9:** Complexity and inference-time comparison of the evaluated CNN models.

Model	Parameters	Parameters (K)	Model Size (MB)	Inference Time (ms/Segment)	Inference Std (ms)	Throughput (Segments/s)
Gated Fusion CNN	204,035	204.035	0.7783	0.3297	0.0939	3033.1319
STFT + 2D-CNN Early Fusion	110,755	110.755	0.4225	0.3556	0.1335	2812.1694
Dual-Branch Feature Fusion	154,755	154.755	0.5903	0.4121	0.2949	2426.5545
1D-CNN Early Fusion	73,891	73.891	0.2819	0.5835	0.2579	1713.7462
STFT + 2D-CNN Vibration	110,467	110.467	0.4214	0.5900	0.0632	1694.9892
1D-CNN Vibration	73,603	73.603	0.2808	0.9409	0.3018	1062.8335

**Table 10:** Bearing-code-disjoint validation results for the two strongest raw temporal models.

Model	Runs	Mean Accuracy	Std Accuracy	Mean Macro-F1	Std Macro-F1	Min Macro-F1	Mean Worst-Class F1	Min Worst-Class F1	Robustness Score	Parameters
1D-CNN Vibration	40	0.6114	0.2102	0.5616	0.2500	0.1678	0.3212	0.0000	0.3116	73,603
1D-CNN Early Fusion	40	0.6040	0.2034	0.5485	0.2443	0.1670	0.2863	0.0000	0.3042	73,891

**Table 11:** Paired statistical comparison under bearing-code-disjoint validation.

Comparison	Paired Runs	Mean Difference	Std Difference	Median Difference	Wilcoxon Statistic	*p*-Value	Bootstrap 95% CI
1D-CNN Early Fusion − 1D-CNN Vibration	40	−0.0130	0.0664	−0.0104	314.0	0.2016	[−0.0336, 0.0069]

**Table 12:** Measurement-wise classical machine-learning baseline summary.

Feature Set	Model	Runs	Mean Accuracy	Std Accuracy	Mean Macro-F1	Std Macro-F1	Min Macro-F1	Mean Worst-Class F1	Robustness Score
Vibration + Current features	XGBoost	5	0.9935	0.0028	0.9937	0.0028	0.9935	0.9918	0.9909
Vibration + Current features	Extra Trees	5	0.9896	0.0043	0.9901	0.0040	0.9876	0.9852	0.9861
Vibration + Current features	Random Forest	5	0.9875	0.0065	0.9892	0.0062	0.9832	0.9854	0.9830
Vibration features	XGBoost	5	0.9690	0.0084	0.9685	0.0087	0.9611	0.9575	0.9598
Vibration features	Random Forest	5	0.9674	0.0138	0.9678	0.0136	0.9662	0.9606	0.9542
Vibration features	Extra Trees	5	0.9651	0.0092	0.9667	0.0093	0.9645	0.9567	0.9574
Vibration + Current features	Linear SVM	5	0.9043	0.0000	0.9052	0.0000	0.9052	0.8910	0.9052
Vibration features	Linear SVM	5	0.8662	0.0000	0.8645	0.0000	0.8645	0.8465	0.8645

**Table 13:** Operating-condition holdout classical machine-learning baseline summary.

Feature Set	Model	Runs	Mean Accuracy	Std Accuracy	Mean Macro-F1	Std Macro-F1	Min Macro-F1	Mean Worst-Class F1	Min Worst-Class F1	Robustness Score
Vibration features	XGBoost	20	0.8584	0.1531	0.8582	0.1542	0.5846	0.8016	0.4193	0.7040
Vibration + Current features	XGBoost	20	0.8563	0.1695	0.8514	0.1788	0.5454	0.7876	0.3581	0.6726
Vibration + Current features	Extra Trees	20	0.8490	0.1655	0.8416	0.1785	0.5368	0.7570	0.2339	0.6632
Vibration features	Random Forest	20	0.8439	0.1835	0.8364	0.1954	0.4891	0.7573	0.2402	0.6411
Vibration features	Extra Trees	20	0.8413	0.1906	0.8353	0.2005	0.4862	0.7560	0.2349	0.6347
Vibration + Current features	Random Forest	20	0.8421	0.1832	0.8365	0.1990	0.4935	0.7519	0.2048	0.6356
Vibration features	Linear SVM	20	0.7332	0.1672	0.7298	0.1675	0.4900	0.6927	0.4683	0.5623
Vibration + Current features	Linear SVM	20	0.6278	0.2012	0.5973	0.2260	0.3700	0.4264	0.0000	0.3713

## Data Availability

The original Paderborn bearing dataset analyzed in this study is publicly available through the Paderborn University Bearing Data Center. The processed segment-level metadata, measurement-wise split assignments, excluded-file log, bearing-code-disjoint validation results, and classical baseline result tables generated during the current study are provided as Supplementary Machine-Readable Files and are also available from the corresponding author upon reasonable request.

## Supplementary Materials

The following supporting information can be downloaded at: https://www.mdpi.com/article/10.3390/s26123829/s1, Table S1a: Complete condition-holdout results of all evaluated models; Table S1b: Macro-F1 and robustness summary across condition-holdout tests; Table S2a: Summary of selected bearing codes by diagnostic class; Table S2b: Per-bearing and per-condition measurement-file and segment counts; Table S2c: Machine-readable supplementary data files provided for reproducibility; Table S3a: Bearing-code-disjoint validation summary; Table S3b: Scenario-wise bearing-code-disjoint validation summary; Table S3c: Paired statistical test for bearing-code-disjoint validation; Table S4a: Classical measurement-wise baseline summary; Table S4b: Classical condition-holdout baseline summary. The supplementary package also includes the following machine-readable CSV files: paderborn_medium_3class_4096_nooverlap_10seg_metadata.csv, paderborn_medium_3class_4096_nooverlap_10seg_errors.csv, bearing_code_holdout_scenarios.csv, bearing_code_disjoint_validation_results.csv, classical_measurementwise_results.csv, and classical_condition_holdout_results.csv.
