# Supplementary material for: IoT-Driven Robust Bearing Fault Diagnosis for Induction Motors Under Operating-Condition Shift
Source: Sensors (Basel). 2026 Jun 16;26(12):3829. doi: 10.3390/s26123829 (PMC13306693; doi:10.3390/s26123829)
Supplement: Supplementary file 1 [file sensors-26-03829-s001.zip › sensors-4344250-supplementary.pdf]

# Supplementary Materials

## Extended validation results, Paderborn subset composition, bearing-code-disjoint analysis, and classical baselines

This Supplementary File provides the extended numerical results, subset-composition details, bearing-code-disjoint validation summaries, and classical machine-learning baseline results supporting the revised manuscript. Tables are numbered with the prefix S to distinguish them from the main manuscript Tables.

### Supplementary Table S1a. Complete condition-holdout results of all evaluated models.

This Table reports the complete condition-holdout results for all evaluated diagnostic models across the four unseen operating conditions. The reported metrics include test accuracy, test loss, macro-precision, macro-recall, Macro-F1, weighted F1, and training time.

| Test condition | Model | Test accuracy | Test loss | Macro-precision | Macro-recall | Macro-F1 | Weighted F1 | Training time (s) |
|---|---|---|---|---|---|---|---|---|
| N09_M07_F10 | 1D-CNN Vibration | 0.6948 | 1.3373 | 0.7243 | 0.7063 | 0.7103 | 0.6957 | 41.2807 |
| N09_M07_F10 | 1D-CNN Current | 0.3655 | 1.0897 | 0.6929 | 0.3353 | 0.1821 | 0.1979 | 39.5890 |
| N09_M07_F10 | 1D-CNN Early Fusion | 0.7316 | 1.1907 | 0.7600 | 0.7399 | 0.7474 | 0.7346 | 42.2116 |
| N09_M07_F10 | STFT + 2D-CNN Vibration | 0.5805 | 2.0870 | 0.5843 | 0.6015 | 0.5799 | 0.5676 | 37.9648 |
| N09_M07_F10 | STFT + 2D-CNN Early Fusion | 0.6311 | 1.8682 | 0.6271 | 0.6481 | 0.6246 | 0.6155 | 43.3613 |
| N09_M07_F10 | Dual-Branch Feature Fusion | 0.7620 | 1.1368 | 0.7867 | 0.7680 | 0.7756 | 0.7651 | 73.9444 |
| N09_M07_F10 | Gated Fusion CNN | 0.6970 | 1.3498 | 0.6979 | 0.7084 | 0.7012 | 0.6949 | 74.3653 |
| N15_M01_F10 | 1D-CNN Vibration | 0.9287 | 0.1865 | 0.9399 | 0.9205 | 0.9266 | 0.9284 | 41.7817 |
| N15_M01_F10 | 1D-CNN Current | 0.3622 | 1.0946 | 0.1207 | 0.3333 | 0.1773 | 0.1926 | 20.6830 |
| N15_M01_F10 | 1D-CNN Early Fusion | 0.9036 | 0.3007 | 0.9177 | 0.8951 | 0.9016 | 0.9027 | 43.3640 |
| N15_M01_F10 | STFT + 2D-CNN Vibration | 0.8984 | 0.2899 | 0.9135 | 0.8901 | 0.8973 | 0.8982 | 38.9360 |
| N15_M01_F10 | STFT + 2D-CNN Early Fusion | 0.8984 | 0.3420 | 0.9138 | 0.8894 | 0.8958 | 0.8971 | 42.9007 |
| N15_M01_F10 | Dual-Branch Feature Fusion | 0.7237 | 1.8938 | 0.8182 | 0.6721 | 0.6093 | 0.6478 | 73.4139 |
| N15_M01_F10 | Gated Fusion CNN | 0.5640 | 0.9724 | 0.7049 | 0.5340 | 0.5215 | 0.5386 | 38.9073 |
| N15_M07_F04 | 1D-CNN Vibration | 0.8705 | 0.3526 | 0.8930 | 0.8629 | 0.8706 | 0.8706 | 34.2010 |
| N15_M07_F04 | 1D-CNN Current | 0.3636 | 1.1021 | 0.1212 | 0.3333 | 0.1778 | 0.1939 | 21.2105 |
| N15_M07_F04 | 1D-CNN Early Fusion | 0.8770 | 0.2604 | 0.8964 | 0.8707 | 0.8777 | 0.8774 | 43.8611 |
| N15_M07_F04 | STFT + 2D-CNN Vibration | 0.9093 | 0.2142 | 0.9123 | 0.9067 | 0.9085 | 0.9099 | 41.2836 |
| N15_M07_F04 | STFT + 2D-CNN Early Fusion | 0.9341 | 0.1434 | 0.9308 | 0.9354 | 0.9323 | 0.9347 | 43.3762 |
| N15_M07_F04 | Dual-Branch Feature Fusion | 0.8898 | 0.2522 | 0.8871 | 0.8857 | 0.8863 | 0.8896 | 73.0883 |
| N15_M07_F04 | Gated Fusion CNN | 0.8573 | 0.3205 | 0.8564 | 0.8656 | 0.8586 | 0.8564 | 87.2925 |
| N15_M07_F10 | 1D-CNN Vibration | 0.9318 | 0.1582 | 0.9457 | 0.9220 | 0.9288 | 0.9317 | 41.7332 |
| N15_M07_F10 | 1D-CNN Current | 0.3636 | 1.0935 | 0.1212 | 0.3333 | 0.1778 | 0.1939 | 21.8355 |
| N15_M07_F10 | 1D-CNN Early Fusion | 0.9150 | 0.2084 | 0.9357 | 0.9033 | 0.9115 | 0.9150 | 44.2287 |
| N15_M07_F10 | STFT + 2D-CNN Vibration | 0.9516 | 0.0928 | 0.9551 | 0.9444 | 0.9483 | 0.9514 | 40.6478 |

| Test condition | Model | Test accuracy | Test loss | Macro-precision | Macro-recall | Macro-F1 | Weighted F1 | Training time (s) |
|---|---|---|---|---|---|---|---|---|
| N15_M07_F10 | STFT + 2D-CNN Early Fusion | 0.9534 | 0.0954 | 0.9617 | 0.9434 | 0.9490 | 0.9527 | 43.3318 |
| N15_M07_F10 | Dual-Branch Feature Fusion | 0.9025 | 0.2322 | 0.9260 | 0.8956 | 0.9034 | 0.9037 | 72.0866 |
| N15_M07_F10 | Gated Fusion CNN | 0.9109 | 0.2253 | 0.9322 | 0.8964 | 0.9046 | 0.9098 | 84.6923 |

**Supplementary Table S1b. Macro-F1 and robustness summary across condition-holdout tests.**

This Table summarizes the Macro-F1 values of all evaluated models across the four condition-holdout tests, together with mean Macro-F1, standard deviation of Macro-F1, worst-case Macro-F1, and robustness score.

| Test condition / Metric | 1D-CNN Vibration | 1D-CNN Current | 1D-CNN Early Fusion | STFT + 2D-CNN Vibration | STFT + 2D-CNN Early Fusion | Dual-Branch Feature Fusion | Gated Fusion CNN |
|---|---|---|---|---|---|---|---|
| N09_M07_F10 | 0.7103 | 0.1821 | 0.7474 | 0.5799 | 0.6246 | 0.7756 | 0.7012 |
| N15_M01_F10 | 0.9266 | 0.1773 | 0.9016 | 0.8973 | 0.8958 | 0.6093 | 0.5215 |
| N15_M07_F04 | 0.8706 | 0.1778 | 0.8777 | 0.9085 | 0.9323 | 0.8863 | 0.8586 |
| N15_M07_F10 | 0.9288 | 0.1778 | 0.9115 | 0.9483 | 0.9490 | 0.9034 | 0.9046 |
| **Mean Macro-F1** | 0.8591 | 0.1787 | 0.8595 | 0.8335 | 0.8504 | 0.7937 | 0.7465 |
| **Std Macro-F1** | 0.1028 | 0.0023 | 0.0761 | 0.1705 | 0.1522 | 0.1353 | 0.1734 |
| **Worst Macro-F1** | 0.7103 | 0.1773 | 0.7474 | 0.5799 | 0.6246 | 0.6093 | 0.5215 |
| **Robustness Score** | 0.7563 | 0.1765 | 0.7834 | 0.6630 | 0.6983 | 0.6584 | 0.5731 |

**Note:** The robustness score was calculated as the mean Macro-F1 minus the standard deviation of Macro-F1 across the four condition-holdout tests. A higher robustness score indicates better average diagnostic performance with lower variability across unseen operating conditions.

**Supplementary Table S2a. Summary of selected bearing codes by diagnostic class.**

**Supplementary Tables S2a and S2b report the bearing codes, diagnostic classes, operating conditions, measurement-file counts, and segment counts used to construct the medium Paderborn subset analyzed in this study. The Tables are provided to support reproducibility and to clarify the class composition and operating-condition coverage of the selected subset.**

| Class | Number of bearing codes | Bearing codes | Measurement files | Total segments |
|---|---|---|---|---|
| artificial_damage | 8 | KA01, KA03, KA04, KA05, KA06, KA07, KA08, KA09 | 639 | 6390 |
| healthy | 6 | K001, K002, K003, K004, K005, K006 | 480 | 4800 |
| real_damage | 8 | KI01, KI03, KI04, KI05, KI07, KI08, KI14, KI16 | 640 | 6400 |

**Supplementary Table S2b. Per-bearing and per-condition measurement-file and segment counts.**

This Table provides the detailed per-bearing and per-condition composition of the selected medium Paderborn subset, including measurement-file counts, segments per file, and total segment counts.

| Class | Bearing code | Operating condition | Measurement files | Segments per file | Total segments |
|---|---|---|---|---|---|
| artificial_damage | KA01 | N09_M07_F10 | 20 | 10.0 | 200 |
| artificial_damage | KA01 | N15_M01_F10 | 20 | 10.0 | 200 |
| artificial_damage | KA01 | N15_M07_F04 | 20 | 10.0 | 200 |
| artificial_damage | KA01 | N15_M07_F10 | 20 | 10.0 | 200 |
| artificial_damage | KA03 | N09_M07_F10 | 20 | 10.0 | 200 |
| artificial_damage | KA03 | N15_M01_F10 | 20 | 10.0 | 200 |
| artificial_damage | KA03 | N15_M07_F04 | 20 | 10.0 | 200 |
| artificial_damage | KA03 | N15_M07_F10 | 20 | 10.0 | 200 |
| artificial_damage | KA04 | N09_M07_F10 | 20 | 10.0 | 200 |
| artificial_damage | KA04 | N15_M01_F10 | 20 | 10.0 | 200 |
| artificial_damage | KA04 | N15_M07_F04 | 20 | 10.0 | 200 |
| artificial_damage | KA04 | N15_M07_F10 | 20 | 10.0 | 200 |
| artificial_damage | KA05 | N09_M07_F10 | 20 | 10.0 | 200 |
| artificial_damage | KA05 | N15_M01_F10 | 20 | 10.0 | 200 |
| artificial_damage | KA05 | N15_M07_F04 | 20 | 10.0 | 200 |
| artificial_damage | KA05 | N15_M07_F10 | 20 | 10.0 | 200 |
| artificial_damage | KA06 | N09_M07_F10 | 20 | 10.0 | 200 |
| artificial_damage | KA06 | N15_M01_F10 | 20 | 10.0 | 200 |
| artificial_damage | KA06 | N15_M07_F04 | 20 | 10.0 | 200 |
| artificial_damage | KA06 | N15_M07_F10 | 20 | 10.0 | 200 |
| artificial_damage | KA07 | N09_M07_F10 | 20 | 10.0 | 200 |
| artificial_damage | KA07 | N15_M01_F10 | 20 | 10.0 | 200 |
| artificial_damage | KA07 | N15_M07_F04 | 20 | 10.0 | 200 |
| artificial_damage | KA07 | N15_M07_F10 | 20 | 10.0 | 200 |
| artificial_damage | KA08 | N09_M07_F10 | 20 | 10.0 | 200 |
| artificial_damage | KA08 | N15_M01_F10 | 19 | 10.0 | 190 |
| artificial_damage | KA08 | N15_M07_F04 | 20 | 10.0 | 200 |
| artificial_damage | KA08 | N15_M07_F10 | 20 | 10.0 | 200 |
| artificial_damage | KA09 | N09_M07_F10 | 20 | 10.0 | 200 |
| artificial_damage | KA09 | N15_M01_F10 | 20 | 10.0 | 200 |

| Class | Bearing code | Operating condition | Measurement files | Segments per file | Total segments |
|---|---|---|---|---|---|
| artificial_damage | KA09 | N15_M07_F04 | 20 | 10.0 | 200 |
| artificial_damage | KA09 | N15_M07_F10 | 20 | 10.0 | 200 |
| healthy | K001 | N09_M07_F10 | 20 | 10.0 | 200 |
| healthy | K001 | N15_M01_F10 | 20 | 10.0 | 200 |
| healthy | K001 | N15_M07_F04 | 20 | 10.0 | 200 |
| healthy | K001 | N15_M07_F10 | 20 | 10.0 | 200 |
| healthy | K002 | N09_M07_F10 | 20 | 10.0 | 200 |
| healthy | K002 | N15_M01_F10 | 20 | 10.0 | 200 |
| healthy | K002 | N15_M07_F04 | 20 | 10.0 | 200 |
| healthy | K002 | N15_M07_F10 | 20 | 10.0 | 200 |
| healthy | K003 | N09_M07_F10 | 20 | 10.0 | 200 |
| healthy | K003 | N15_M01_F10 | 20 | 10.0 | 200 |
| healthy | K003 | N15_M07_F04 | 20 | 10.0 | 200 |
| healthy | K003 | N15_M07_F10 | 20 | 10.0 | 200 |
| healthy | K004 | N09_M07_F10 | 20 | 10.0 | 200 |
| healthy | K004 | N15_M01_F10 | 20 | 10.0 | 200 |
| healthy | K004 | N15_M07_F04 | 20 | 10.0 | 200 |
| healthy | K004 | N15_M07_F10 | 20 | 10.0 | 200 |
| healthy | K005 | N09_M07_F10 | 20 | 10.0 | 200 |
| healthy | K005 | N15_M01_F10 | 20 | 10.0 | 200 |
| healthy | K005 | N15_M07_F04 | 20 | 10.0 | 200 |
| healthy | K005 | N15_M07_F10 | 20 | 10.0 | 200 |
| healthy | K006 | N09_M07_F10 | 20 | 10.0 | 200 |
| healthy | K006 | N15_M01_F10 | 20 | 10.0 | 200 |
| healthy | K006 | N15_M07_F04 | 20 | 10.0 | 200 |
| healthy | K006 | N15_M07_F10 | 20 | 10.0 | 200 |
| real_damage | KI01 | N09_M07_F10 | 20 | 10.0 | 200 |
| real_damage | KI01 | N15_M01_F10 | 20 | 10.0 | 200 |
| real_damage | KI01 | N15_M07_F04 | 20 | 10.0 | 200 |
| real_damage | KI01 | N15_M07_F10 | 20 | 10.0 | 200 |
| real_damage | KI03 | N09_M07_F10 | 20 | 10.0 | 200 |
| real_damage | KI03 | N15_M01_F10 | 20 | 10.0 | 200 |

| Class | Bearing code | Operating condition | Measurement files | Segments per file | Total segments |
|---|---|---|---|---|---|
| real_damage | KI03 | N15_M07_F04 | 20 | 10.0 | 200 |
| real_damage | KI03 | N15_M07_F10 | 20 | 10.0 | 200 |
| real_damage | KI04 | N09_M07_F10 | 20 | 10.0 | 200 |
| real_damage | KI04 | N15_M01_F10 | 20 | 10.0 | 200 |
| real_damage | KI04 | N15_M07_F04 | 20 | 10.0 | 200 |
| real_damage | KI04 | N15_M07_F10 | 20 | 10.0 | 200 |
| real_damage | KI05 | N09_M07_F10 | 20 | 10.0 | 200 |
| real_damage | KI05 | N15_M01_F10 | 20 | 10.0 | 200 |
| real_damage | KI05 | N15_M07_F04 | 20 | 10.0 | 200 |
| real_damage | KI05 | N15_M07_F10 | 20 | 10.0 | 200 |
| real_damage | KI07 | N09_M07_F10 | 20 | 10.0 | 200 |
| real_damage | KI07 | N15_M01_F10 | 20 | 10.0 | 200 |
| real_damage | KI07 | N15_M07_F04 | 20 | 10.0 | 200 |
| real_damage | KI07 | N15_M07_F10 | 20 | 10.0 | 200 |
| real_damage | KI08 | N09_M07_F10 | 20 | 10.0 | 200 |
| real_damage | KI08 | N15_M01_F10 | 20 | 10.0 | 200 |
| real_damage | KI08 | N15_M07_F04 | 20 | 10.0 | 200 |
| real_damage | KI08 | N15_M07_F10 | 20 | 10.0 | 200 |
| real_damage | KI14 | N09_M07_F10 | 20 | 10.0 | 200 |
| real_damage | KI14 | N15_M01_F10 | 20 | 10.0 | 200 |
| real_damage | KI14 | N15_M07_F04 | 20 | 10.0 | 200 |
| real_damage | KI14 | N15_M07_F10 | 20 | 10.0 | 200 |
| real_damage | KI16 | N09_M07_F10 | 20 | 10.0 | 200 |
| real_damage | KI16 | N15_M01_F10 | 20 | 10.0 | 200 |
| real_damage | KI16 | N15_M07_F04 | 20 | 10.0 | 200 |
| real_damage | KI16 | N15_M07_F10 | 20 | 10.0 | 200 |

**Note:** Each valid measurement file was divided into 10 non-overlapping segments with a window size of 4096 samples. The artificial-damage class contains 639 valid measurement files because one measurement file, N15_M01_F10_KA08_2.mat, could not be parsed successfully during preprocessing and was excluded. Therefore, the final medium subset contained 17,590 valid segments.

## Supplementary Data Files for Reproducibility

In addition to Supplementary Tables S2a and S2b, the revised supplementary package includes machine-readable CSV files to support reproducibility. These files provide the complete file-level manifest, segment-level metadata, measurement-wise split assignments, condition-holdout split information, and excluded-file information used in the experiments. This format was selected because the complete segment-level manifest contains 17,590 rows and is more suitable for machine-readable supplementary data than for inclusion as a formatted manuscript Table.

**Supplementary Table S2c. Machine-readable supplementary data files provided for reproducibility.**

| File | Description |
|---|---|
| **paderborn_medium_3class_4096_nooverlap_10seg_metadata.csv** | **Segment-level metadata including file name, file path, diagnostic label, label ID, bearing code, operating condition, start sample, end sample, vibration signal name, current signal name, and measurement-wise split assignment.** |
| **paderborn_medium_3class_4096_nooverlap_10seg_errors.csv** | **Excluded or failed files detected during preprocessing, including the excluded artificial-damage file.** |
| **bearing_code_holdout_scenarios.csv** | **Definition of the eight bearing-code-disjoint validation scenarios, including held-out healthy, artificial-damage, and real-damage bearing codes.** |
| **bearing_code_disjoint_validation_results.csv** | **Complete 80-run bearing-code-disjoint validation results for the 1D-CNN Vibration and 1D-CNN Early Fusion models.** |
| **classical_measurementwise_results.csv** | **Complete measurement-wise classical baseline results for all feature sets, classifiers, and seeds.** |
| **classical_condition_holdout_results.csv** | **Complete operating-condition-holdout classical baseline results for all feature sets, classifiers, holdout conditions, and seeds.** |

**Supplementary Table S3a. Bearing-code-disjoint validation summary.**

This Table summarizes the additional bearing-code-disjoint stress test for the two strongest raw temporal models. Each model was evaluated across eight bearing-code holdout scenarios and five random seeds, producing 40 runs per model.

| Model | Runs | Accuracy (mean ± SD) | Macro-F1 (mean ± SD) | Min Macro-F1 | Mean worst-class F1 | Robustness score | Parameters |
|---|---|---|---|---|---|---|---|
| 1D-CNN Vibration | 40 | 0.6114 ± 0.2102 | 0.5616 ± 0.2500 | 0.1678 | 0.3212 | 0.3116 | 73,603 |
| 1D-CNN Early Fusion | 40 | 0.6040 ± 0.2034 | 0.5485 ± 0.2443 | 0.1670 | 0.2863 | 0.3042 | 73,891 |

**Supplementary Table S3b. Scenario-wise bearing-code-disjoint validation summary.**

This Table reports the scenario-wise mean performance of the two raw temporal models under bearing-code-disjoint validation. Each row summarizes five random seeds for the corresponding scenario and model.

| Scenario | Heldout codes | Model | Runs | Mean Acc. | Mean Macro-F1 | SD Macro-F1 | Min Macro-F1 | Mean worst-class F1 |
|---|---|---|---|---|---|---|---|---|
| BC_HOLDOUT_1 | K001, KA01, KI01 | 1D-CNN Early Fusion | 5 | 0.5342 | 0.4622 | 0.0569 | 0.4183 | 0.0057 |
| BC_HOLDOUT_1 | K001, KA01, KI01 | 1D-CNN Vibration | 5 | 0.5143 | 0.4580 | 0.0368 | 0.4224 | 0.0268 |
| BC_HOLDOUT_2 | K002, KA03, KI03 | 1D-CNN Early Fusion | 5 | 0.3342 | 0.1711 | 0.0055 | 0.1670 | 0.0000 |
| BC_HOLDOUT_2 | K002, KA03, KI03 | 1D-CNN Vibration | 5 | 0.3333 | 0.1697 | 0.0023 | 0.1678 | 0.0000 |

| Scenario | Heldout codes | Model | Runs | Mean Acc. | Mean Macro-F1 | SD Macro-F1 | Min Macro-F1 | Mean worst-class F1 |
|---|---|---|---|---|---|---|---|---|
| BC_HOLDOUT_3 | K003, KA04, KI04 | 1D-CNN Early Fusion | 5 | 0.4308 | 0.4149 | 0.0540 | 0.3584 | 0.0165 |
| BC_HOLDOUT_3 | K003, KA04, KI04 | 1D-CNN Vibration | 5 | 0.4124 | 0.4054 | 0.0308 | 0.3734 | 0.0387 |
| BC_HOLDOUT_4 | K004, KA05, KI05 | 1D-CNN Early Fusion | 5 | 0.6860 | 0.6076 | 0.0729 | 0.5669 | 0.1570 |
| BC_HOLDOUT_4 | K004, KA05, KI05 | 1D-CNN Vibration | 5 | 0.6916 | 0.6223 | 0.0453 | 0.5600 | 0.2038 |
| BC_HOLDOUT_5 | K005, KA06, KI07 | 1D-CNN Early Fusion | 5 | 0.7862 | 0.7887 | 0.0357 | 0.7410 | 0.7357 |
| BC_HOLDOUT_5 | K005, KA06, KI07 | 1D-CNN Vibration | 5 | 0.7488 | 0.7514 | 0.0272 | 0.7260 | 0.7022 |
| BC_HOLDOUT_6 | K006, KA07, KI08 | 1D-CNN Early Fusion | 5 | 0.6924 | 0.6671 | 0.0970 | 0.5230 | 0.4673 |
| BC_HOLDOUT_6 | K006, KA07, KI08 | 1D-CNN Vibration | 5 | 0.7764 | 0.7641 | 0.0431 | 0.6999 | 0.6486 |
| BC_HOLDOUT_7 | K001, KA08, KI14 | 1D-CNN Early Fusion | 5 | 0.9439 | 0.9428 | 0.0517 | 0.8588 | 0.9075 |
| BC_HOLDOUT_7 | K001, KA08, KI14 | 1D-CNN Vibration | 5 | 0.9670 | 0.9667 | 0.0228 | 0.9300 | 0.9493 |
| BC_HOLDOUT_8 | K002, KA09, KI16 | 1D-CNN Early Fusion | 5 | 0.4243 | 0.3336 | 0.0363 | 0.2900 | 0.0000 |
| BC_HOLDOUT_8 | K002, KA09, KI16 | 1D-CNN Vibration | 5 | 0.4473 | 0.3551 | 0.0791 | 0.2907 | 0.0000 |

**Supplementary Table S3c. Paired statistical test for bearing-code-disjoint validation.**

This Table reports the paired statistical comparison between 1D-CNN Early Fusion and 1D-CNN Vibration across 40 paired bearing-code-disjoint runs.

| Comparison | Paired runs | Mean difference | Median difference | Wilcoxon p-value | Bootstrap 95% CI |
|---|---|---|---|---|---|
| Early Fusion - Vibration | 40 | -0.0130 | -0.0104 | 0.2016 | [-0.0336, 0.0069] |

**Supplementary Table S4a. Classical measurement-wise baseline summary.**

This Table summarizes the classical machine-learning baselines under measurement-wise validation. The vibration feature set contains 33 handcrafted features, while the vibration-current feature set contains 66 features.

| Feature set | Model | Runs | Accuracy (mean ± SD) | Macro-F1 (mean ± SD) | Min Macro-F1 | Mean worst-class F1 | Min worst-class F1 | Robustness score |
|---|---|---|---|---|---|---|---|---|
| Vibration+Current | XGBoost | 5 | 0.9935 ± 0.0028 | 0.9937 ± 0.0028 | 0.9935 | 0.9918 | 0.9914 | 0.9909 |
| Vibration+Current | Extra Trees | 5 | 0.9896 ± 0.0043 | 0.9901 ± 0.0040 | 0.9876 | 0.9852 | 0.9846 | 0.9861 |
| Vibration+Current | Random Forest | 5 | 0.9875 ± 0.0065 | 0.9892 ± 0.0062 | 0.9832 | 0.9854 | 0.9842 | 0.9830 |

| Feature set | Model | Runs | Accuracy (mean ± SD) | Macro-F1 (mean ± SD) | Min Macro-F1 | Mean worst-class F1 | Min worst-class F1 | Robustness score |
|---|---|---|---|---|---|---|---|---|
| Vibration | XGBoost | 5 | 0.9690 ± 0.0084 | 0.9685 ± 0.0087 | 0.9611 | 0.9575 | 0.9562 | 0.9598 |
| Vibration | Random Forest | 5 | 0.9674 ± 0.0138 | 0.9678 ± 0.0136 | 0.9662 | 0.9606 | 0.9591 | 0.9542 |
| Vibration | Extra Trees | 5 | 0.9651 ± 0.0092 | 0.9667 ± 0.0093 | 0.9645 | 0.9567 | 0.9552 | 0.9574 |
| Vibration+Current | Linear SVM | 5 | 0.9043 ± 0.0000 | 0.9052 ± 0.0000 | 0.9052 | 0.8910 | 0.8910 | 0.9052 |
| Vibration | Linear SVM | 5 | 0.8662 ± 0.0000 | 0.8645 ± 0.0000 | 0.8645 | 0.8465 | 0.8465 | 0.8645 |

**Supplementary Table S4b. Classical condition-holdout baseline summary.**

This Table summarizes the classical machine-learning baselines under operating-condition-holdout validation. Each row summarizes 20 runs, corresponding to four holdout conditions and five random seeds.

| Feature set | Model | Runs | Accuracy (mean ± SD) | Macro-F1 (mean ± SD) | Min Macro-F1 | Mean worst-class F1 | Min worst-class F1 | Robustness score |
|---|---|---|---|---|---|---|---|---|
| Vibration | XGBoost | 20 | 0.8584 ± 0.1531 | 0.8582 ± 0.1542 | 0.5846 | 0.8016 | 0.4193 | 0.7040 |
| Vibration+Current | XGBoost | 20 | 0.8563 ± 0.1695 | 0.8514 ± 0.1788 | 0.5454 | 0.7876 | 0.3581 | 0.6726 |
| Vibration+Current | Extra Trees | 20 | 0.8490 ± 0.1655 | 0.8416 ± 0.1785 | 0.5368 | 0.7570 | 0.2339 | 0.6632 |
| Vibration | Random Forest | 20 | 0.8439 ± 0.1835 | 0.8364 ± 0.1954 | 0.4891 | 0.7573 | 0.2402 | 0.6411 |
| Vibration | Extra Trees | 20 | 0.8413 ± 0.1906 | 0.8353 ± 0.2005 | 0.4862 | 0.7560 | 0.2349 | 0.6347 |
| Vibration+Current | Random Forest | 20 | 0.8421 ± 0.1832 | 0.8365 ± 0.1990 | 0.4935 | 0.7519 | 0.2048 | 0.6356 |
| Vibration | Linear SVM | 20 | 0.7332 ± 0.1672 | 0.7298 ± 0.1675 | 0.4900 | 0.6927 | 0.4683 | 0.5623 |
| Vibration+Current | Linear SVM | 20 | 0.6278 ± 0.2012 | 0.5973 ± 0.2260 | 0.3700 | 0.4264 | 0.0000 | 0.3713 |
